# COLLAGENE enables privacy-aware federated and collaborative genomic data analysis

**DOI:** 10.1186/s13059-023-03039-z

**Published:** 2023-09-11

**Authors:** Wentao Li, Miran Kim, Kai Zhang, Han Chen, Xiaoqian Jiang, Arif Harmanci

**Affiliations:** 1 Center for Secure Artificial Intelligence For hEalthcare (SAFE), D. Bradley McWilliams School of Biomedical Informatics, University of Texas Health Science Center, Houston, TX 77030 USA; 2 Department of Mathematics, Department of Computer Science, Hanyang University, Seoul, 04763 Republic of Korea; 3 Research Institute for Convergence of Basic Science, Hanyang University, Seoul, 04763 Republic of Korea; 4 Bio-BigData Center, Hanyang Institute of Bioscience and Biotechnology, Hanyang University, Seoul, 04763 Republic of Korea; 5 Human Genetics Center, Department of Epidemiology, Human Genetics and Environmental Sciences, School of Public Health, The University of Texas Health Science Center at Houston, Houston, TX 77030 USA; 6 Center for Precision Health, D. Bradley McWilliams School of Biomedical Informatics, The University of Texas Health Science Center at Houston, Houston, TX 77030 USA

**Keywords:** Collaborative analysis, Federated model training, Genomic data privacy, Data security

## Abstract

Growing regulatory requirements set barriers around genetic data sharing and collaborations. Moreover, existing privacy-aware paradigms are challenging to deploy in collaborative settings. We present COLLAGENE, a tool base for building secure collaborative genomic data analysis methods. COLLAGENE protects data using shared-key homomorphic encryption and combines encryption with multiparty strategies for efficient privacy-aware collaborative method development. COLLAGENE provides ready-to-run tools for encryption/decryption, matrix processing, and network transfers, which can be immediately integrated into existing pipelines. We demonstrate the usage of COLLAGENE by building a practical federated GWAS protocol for binary phenotypes and a secure meta-analysis protocol. COLLAGENE is available at https://zenodo.org/record/8125935.

**Supplementary Information:**

The online version contains supplementary material available at 10.1186/s13059-023-03039-z.

## Background

The accumulation of genetic and biomedical data is promising for advancing translational approaches [1–3], diagnosis and management of diseases [4], and improving the quality of life for patients and individuals at risk [5]. As the high-throughput data acquisition cost is decreasing (DNA sequencing, EHR databases, high-throughput phenotyping technologies), several major challenges are rising around governance [6] and the protection of individual-level data [7–9].

As open data sharing is more incentivized by funders and the scientific community [10–12], the challenges around data management become more pronounced [13]. Particularly, data sharing requires collaboration among multiple institutions that would like to share data to increase the statistical power of the models that can detect more intricate patterns within complex biomedical data, e.g., genome-wide association studies among sites [14–16]. However, collaborations can only be possible if the regulations around data sharing are adhered to [17–21]. Policy-makers around the world are pushing for more data protection with almost zero tolerance on sharing, reuse, and incidental reporting of individual-level datasets, even at the encrypted and pseudo-anonymized level, e.g., GDPR [22–25]. Federated model development [26–28] is a promising approach for this purpose: Each site locally processes sensitive dataset and share only intermediate model updates with other sites. Since the sites only exchange aggregated data, these approaches provide a certain level of protection [29–33]. However, previous studies demonstrated that even summary statistic-level data may impose privacy risks on participants [34–38].

Privacy-aware collaboration has been an extensive and active field of research. Of particular interest is the horizontal partitioning of data, where each site harbors data for different subjects with similar features across sites [39–42]. Libraries such as FedML [43] and PySyft [44, 45] provide programming interfaces to build machine learning models using a federated approach. Specifically, PySyft integrates privacy and security into the model-building framework and it is targeted for building deep learning applications. Differential privacy-based data protection has been utilized in many previous methods. For example, *TensorFlow-Privacy* [46] makes use of differential privacy (DP) [47] to protect intermediate summary statistics in the training of deep learning models. One of the main challenges of the DP-based approaches is the selection of the privacy budget and balancing noisy data utility for ensuring model accuracy [48]. A related approach, HyFED [49], provides means to use masking intermediate data with noise to protect these data while building more custom approaches and mitigating privacy issues. However, it is necessary to make use of two sites, aggregator and compensator, which must be included in the computation protocol for removing noise from the data. Any collusions between these sites and other sites will leak intermediate or individual data.

Homomorphic encryption (HE) [50, 51] provides strong guarantees for the protection of data because the data is encrypted once and is not decrypted even while it is being analyzed. While HE does not provide straightforward collaboration among multiple parties, multi-key [52, 53] approaches and threshold-HE [54] approaches are developed for enabling the data encryption using single or multiple keys and using collective decryption among collaborating sites. Lattigo [55], Palisade [56, 57], and MK-TFHE [58] are libraries that provide the programming interfaces to use threshold and multi-key-based collaborative tool development. Similar to HE, multiparty computation (MPC) [59] provides the frameworks for developing provably secure data analysis approaches with the added benefit of collaboration among sites. Two recent MPC-based efforts for directly compiling code to be runnable in MPC-based primitives (MPC-SoK [60]) and for building MPC-based pipelines (Sequre [61]) can benefit more advanced users because these approaches use specialized programming languages. One of the limiting factors around MPC is that it relies on large data transfers, which may be a limiting factor for their widespread usage when network bandwidth cost is not negligible, e.g., costs for data transfer out of the cloud. Indeed, several previous methods demonstrated that network bandwidth and cost of storage/transfer is the limiting factor for the algorithm efficiency in collaborative studies [62–64]. The most promising approaches are hybrid frameworks that combine HE, MPC, and DP to adhere to the privacy requirements of the local regulations while algorithmic efficiency is satisfied.

Another major challenge is usability. For instance, in the homomorphic encryption domain, there are numerous libraries such as SEAL [65] and TFHE [66] that provide single-key encryption functionalities but they require a working understanding of homomorphic encryption and are not immediately usable in collaborative and federated cases. While these libraries are accessible to advanced users, they are not immediately accessible to general users, for whom there is a steep learning curve for parameter selection, algorithm conversion, and implementation of distributed and privacy-preserving methods.

To overcome these challenges of development and integration, we developed COLLAGENE, a library of tools and services that enable collaborative biomedical data analysis. COLLAGENE makes use of HE as the main means for data protection using the primitives as implemented by the SEAL library. The collaboration is achieved using a threshold-HE system that implements the secret key sharing that was originally proposed by Asharov [67]. COLLAGENE integrates components of MPC, HE, and matrix masking that is motivated by matrix-level differential privacy [68, 69] for performing complex operations (e.g., matrix inversion) efficiently while preserving privacy. COLLAGENE provides ready-to-run implementations for encryption, collective decryption, matrix masking, a suite of secure matrix arithmetic operations, and network file input/output tools for sharing encrypted intermediate datasets among collaborating sites. These tools can be immediately run and integrated into existing pipelines for developing new collaborative analysis protocols or conversion of existing methods into a secure implementation.

Compared to the low-level implementations such as Palisade, and Lattigo, which require high level of expertise, COLLAGENE is more application-oriented (as command line tools) to enable easier development and deployment of collaborative tools. Compared to the higher-level libraries with security integration such as PySyft and FedML that specifically focus on machine learning-based applications, COLLAGENE provides an alternative that can be used for building custom analysis protocols in biomedical informatics community.

For demonstrating the usage of COLLAGENE, we provide examples of encryption, collaborative decryption, and matrix arithmetic operations. As separate use cases, we demonstrate the usage of COLLAGENE’s functionality by implementing genome-wide association testing and meta-analysis for binary traits using a generalized linear model. We present an approach that adopts the efficient 2-step variant scoring of the GMMAT [70] algorithm. We modify GMMAT’s score test to perform a practical privacy-preserving GWAS among multiple sites and demonstrate the usage of COLLAGENE’s tools for converting an existing method into a secure federated approach. COLLAGENE’s command line tools aim at increasing accessibility to secure analysis methods and they can be seamlessly integrated into existing analysis pipelines.

## Results

We first present COLLAGENE’s secure collaboration framework and present the use cases of COLLAGENE framework for federated GWAS on binary phenotypes and meta-analysis of binary traits.

### Collaborative analysis framework

#### Cloud-based key sharing service by *KeyMaker*

**Fig. 1:**
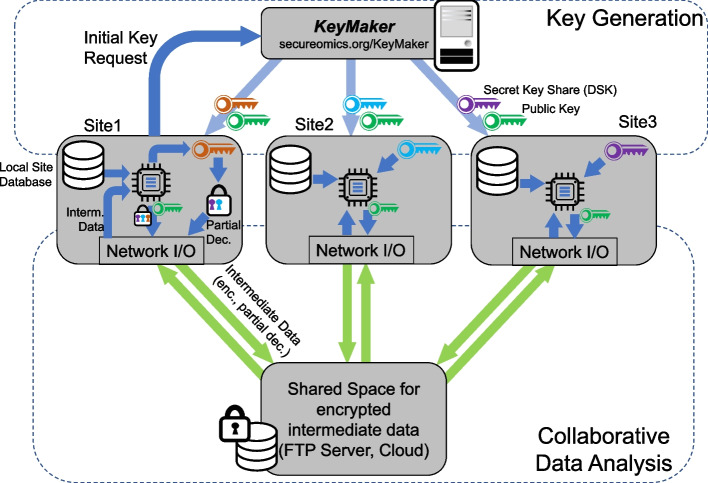
Illustration of the collaborative analysis framework that COLLAGENE implements. Three sites are shown as an example. The sites (rounded rectangles), which host their local datasets, initiate key request from *KeyMaker*. After keys are generated, each site download and decrypt their key shares. Sites also have a common encryption (i.e., public key, green). Next, sites setup a shared space on an FTP server or on the cloud, e.g., AWS S3 bucket. According to the agreed protocol, each sites process their local data using COLLAGENE’s tools and upload encrypted intermediate results to the shared space. Sites download intermediate results from other sites and process these for the next iteration. After all the iterative analysis steps are complete, the sites perform collective decryption as specified by the agreed protocol. Note that *KeyMaker* only participates in the key generation and does not (and should not) take part in data analysis protocol

The collaborative analysis with COLLAGENE makes use of a common public key and usage of secret key shares that are distributed to each collaborating site (Fig. 1). This approach relies on the composability of the secret keys in ring-learning-with-errors schemes. This technique was first proposed by Asharov et al. and has been adopted by numerous other approaches. COLLAGENE relies on a central key sharing service (*KeyMaker*) that generates and shares secret keys among sites, which is publicly available at https://www.secureomics.org/KeyMaker. *KeyMaker* generates a master key and utilizes a noise addition step to “share” it among sites (Methods). The master secret key is not shared with any of the sites and is discarded after keys are generated. In addition to secret key sharing, *KeyMaker* generates the common public key, relinearization keys, and Galois keys, which are necessary for performing HE operations on encrypted data, e.g., secure matrix arithmetic. *KeyMaker* also generates a symmetric encryption key that can be used for tasks such as the encryption of partially decrypted datasets. After keys are generated, the sites download all the keys and independently perform their calculations. *KeyMaker* does not partake in the data analysis. Unlike other approaches that rely on online algorithms that require all sites to be up and running, the usage of a central service for key sharing decreases the workload of generating the shared keys.

#### Secure matrix processing library

**Fig. 2:**
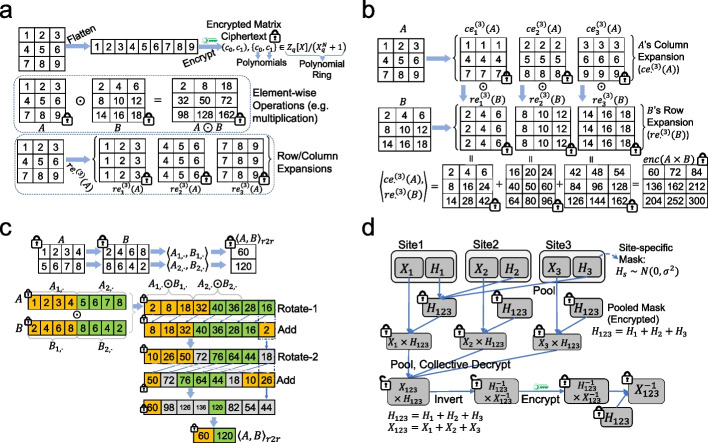
Matrix arithmetic operations supported by COLLAGENE. **a** A matrix is flattened into an array and encrypted using the public key. Encryption output a series of ciphertexts, each of which contains a pair of polynomials in the ring ${Z}_{q}\left[X\right]/\left({X}_{q}^{N}+1\right)$, details of which are encapsulated by COLLAGENE. Elementwise multiplication is illustrated between $3\times 3$ matrices (middle). Row expansion of a matrix is illustrated for a $3\times 3$ matrix (bottom). **b** Multiplication of two matrices ($A$ and $B$) is illustrated using the column and row expansions, denoted by ${ce}_{\cdot }^{(3)}(A)$, and ${re}_{\cdot }^{(3)}(B)$. Multiplication is calculated as the inner product of the expansion matrices, denoted by $\langle {ce}_{\cdot }^{\left(3\right)}\left(A\right),{re}_{\cdot }^{\left(3\right)}\left(B\right)\rangle$. **c** Secure row-row inner product of two matrices by shift-and-add operations. Given $A$ and $B$ matrices of size 2 × 4, row-row inner product is a $2\times 1$ vector whose entries are the inner products of rows of $A$ and $B$ (top). To calculate the inner products, the ciphertext that corresponds to the elementwise multiplication of the matrices ($A\odot B$) is shifted and added recursively (Bottom). The flattened representations of matrices stored in the ciphertexts are colored to indicate rows of $A$ and $B$. At each step, the inner product ciphertext is circularly rotated using Galois keys and added to the current ciphertext. The rotations are depicted by arrows to show the rotated entries. Gray shaded entries indicate unused entries. After 2 rotations, the row-row inner products for each row are stored in orange and green entries. These entries are masked and copied to the final row-row inner product ciphertext, ${\langle A,B\rangle }_{r2r}$. **d** Collaborative matrix inversion protocol that utilizes matrix masking to calculate the inverse of a matrix. Three sites would like to invert the summation of local matrices ${X}_{1}$, $X$, and ${X}_{3}$ that stores sensitive data. Each site first generates a masking matrix ${M}_{1}$, ${M}_{2}$, ${M}_{3}$, then encrypt them and upload to the shared space. Next, sites locally pool the mask matrices ${M}_{123}={M}_{1}+{M}_{2}+{M}_{3}$ to generate the collective encrypted mask. Sites locally multiply the collective mask with their matrix, i.e., site-1 securely multiplies ${X}_{1}\times {M}_{123}$, and upload to the shared space. Each site downloads the masked matrices and pools the matrices to calculate ${X}_{123}\times {M}_{123}$, which is still encrypted. The sites collectively decrypt this matrix, then locally invert the decrypted matrix, which yields ${M}_{123}^{-1}\times {X}_{123}$. Sites finally multiply the inverted matrix on the left with ${M}_{123}$, which results in the ${X}_{123}^{-1}$. The row and column expansions are not shown for the sake of simplicity

The second component of COLLAGENE is the set of standalone command line tools and libraries for processing matrix-formatted datasets. Matrix-based data representation is adopted since most tools in bioinformatics are based on matrix algebra. COLLAGENE implements secure matrix arithmetic operations such as matrix additions, subtractions (Fig. 2a), multiplications (Fig. 2b), and inner products (Fig. 2c) using row and column expansions of matrices, and efficient inner product operators (Methods).

Additionally, COLLAGENE implements two operations necessary for building collaborative and federated algorithms: First is the collaborative decryption of datasets using the secret key shares at each site (Methods). Collaborative decryption is necessary for decrypting matrices (e.g., final results or intermediate statistics) that are encrypted by the public key generated by *KeyMaker*. Given a ciphertext (e.g., encrypted matrix data) that the sites would like to decrypt, each site uses their secret key share to partially decrypt the ciphertext. These partially decrypted data matrices are shared among sites. After each site retrieves the partial decryptions from all other sites, they pool the partial decryptions and obtain the fully decrypted matrix data. This step is implemented into COLLAGENE’s command line tool base so that users can seamlessly integrate them into their pipelines.

The second operation is “matrix masking” which enables the development of multiparty-type protocols. Matrix masking refers to adding noise to matrices in the encrypted domain (additive or multiplicative noise) to hide the underlying data using preset noise levels. In turn, the masked matrix can be decrypted collectively without leakage of sensitive information. This is advantageous since complex operations (e.g., matrix inversions) can be performed on the decrypted masked matrices in the plaintext domain. After the masked matrix is processed in the plaintext domain, the matrix can be re-encrypted, and the mask can be removed appropriately (i.e., additively, or multiplicatively). This process is advantageous since it accomplishes two goals simultaneously, namely, a complex step is efficiently performed, and the matrix is encrypted into a “fresh” ciphertext, and it can be operated on further in the secure domain. This decreases the effective multiplicative depth of the protocol and the storage and CPU requirements (Fig. 2d).

An important aspect of matrix masking is how much masking noise should be added to the matrices before collective decryption. The studies in matrix data privacy based on DP are helpful to set theoretically provable privacy for setting up the mask matrices. These approaches formulated the appropriate structure and levels of matrix noise for privacy-aware matrix-valued data publishing [68, 69]. It should be noted that matrix masking has been utilized in previous studies for implementing secure protocols [55, 71] but are not available for developers in an easy-to-use manner. Currently, COLLAGENE implements mask matrix generation using Gaussian-valued noise by default that can be used for masking encrypted matrices. We provide examples of using the matrix library for different types of operations. COLLAGENE also provides several options to make it easy to select HE parameters (modulus size, polynomial degrees) before building their pipelines while guaranteeing a certain level of security (e.g., 128-bit).

#### Network communication using shared space

An important component of federated learning frameworks is setting up a secure channel for passing intermediate data among the collaborating sites. By default, COLLAGENE relies on sharing encrypted files from a central storage, i.e., a star-shaped network (Fig. 1). File-based communication among sites simplifies broadcasting data that will be shared with all sites, e.g., partially decrypted matrices. COLLAGENE implements the options for uploading to and downloading from the shared space. Additionally, COLLAGENE includes functions to probe and wait for files to become available in the shared space. By default, an SCP file server, or an Amazon Web Services (AWS) S3 bucket can be used to upload, download, probe, and wait for encrypted files.

### Qualitative comparison with other secure federation frameworks

We first compare COLLAGENE’s functionalities with other frameworks that support the development of secure federation tools. We compiled the existing collaborative data analysis methods from the literature and qualitatively compared these approaches, which is summarized in Table 1. We first divided the approaches with respect to application versus library-level implementation. Overall, COLLAGENE aims at providing a ready-to-be-used implementation of encryption/collective decryption and several matrix operations. Among these methods, Cho et al. [72] implements an MPC-based approach for crowdsourcing of GWAS and TrustGWAS [73] provides outsourcing GWAS implementations using Asharov-type multi-key HE to pool data from all sites at an outsourcing entity (e.g., AWS instance) and perform pooled analysis. These methods are case-specific implementations and do not provide standalone libraries or executables for custom operations. Another approach, sPLINK [74], implements a GWAS method and extends the HyFED [49] scheme that relies on trusted Aggregator and Compensator entities that take a direct role in executing the protocols. HyFED relies upon strict non-collusion of these two entities, which receive sensitive components of the data. In comparison, COLLAGENE does not require an external party to be included in the data processing steps, other than *KeyMaker* which does not take part in the processing of sensitive data.

**Table 1:** Overview of existing libraries and applications. Majority of the approaches are open source with a focus on data protection against honest but-curious (HBC) adversaries. Federated protection is provided using combination of homomorphic encryption (HE), multiparty computation MPC), and differential privacy (DP). Majority of the libraries provide custom API for building applications but limited command line (CLI) tools that can be deployed immediately by users

**Method**	**Link/reference**	**Description**	**Open Source**	**Application library**	**Adversary**	**Federated protection**	**Matrix functions**	**Custom CLI**	**Custom API**
**SEAL**	https://github.com/microsoft/SEAL	C++ implementation of RLWE-based HE schemes	Yes	Library	HBC	None	No	No	Yes
**TenSEAL**	https://github.com/OpenMined/TenSEAL	Python wrapper for SEAL with focus on tensor processing	Yes	Library	HBC	None	No	No	Yes
**HyFED**	https://github.com/TUMAIMED/hyfed	MPC-type privacy framework with Aggregator/Compensator entities for secret sharing	Yes	Library	HBC	MPC	No	No	Yes
**MK-TFHE**	https://github.com/ilachill/MKTFHE	C++ implementation of Multi-Key lattice-based HE	Yes	Library	HBC	HE	No	No	Yes
**Lattigo**	https://github.com/tuneinsight/lattigo	GO implementation of lattice-based multiparty HE schemes	Yes	Library	HBC	HE/MPC	No	No	Yes
**PySyft**	https://github.com/OpenMined/PySyft	Python-based Federated machine learning with secure primitives based on SPDZ Protocol	Yes	Library	HBC	DP/MPC/HE	No	No	Yes
**FedML**	https://github.com/FedMLAI/FedML	Python-based Federated machine learning	Yes	Library	HBC	None	No	No	Yes
**TrustGWAS**	https://github.com/melobio/TrustGWAS	Outsourcing for collaborative GWAS method using Asharov-type key sharing	No	Application	HBC	HE	No	No	No
**Intel HE-Toolkit**	https://github.com/intel/he-toolkit	SEAL/Palisade wrapper with Intel CPI Optimizations	Yes	Library	HBC	None	Yes	No	Yes
**SEQURE**	https://github.com/0xTCG/sequre	MPC-based custom python-like programming interface	Yes	Library	HBC	MPC	No	No	Yes
**MPC GWAS**	https://github.com/hhcho/secure-gwas	MPC-based GWAS method	Yes	Application	HBC	MPC over secure channel	No	No	No
**sPLINK**	https://github.com/TUMAIMED/splink	Privacy-aware GWAS method via HyFED	Yes	Application	HBC	HyFed	No	No	No
**OpenFL**	https://github.com/securefederatedai/openfl	Python-based Federation workflow	Yes	Library	HBC	None	No	No	Yes
**Vantage6**	https://github.com/vantage6/vantage6	Python-based Federated Learning platform with UI	Yes	Application	HBC	None	No	Yes	Yes
**Flower**	https://flower.dev/docs/index.html	Python-based Federated machine learning library	Yes	Library	HBC	MPC	No	No	Yes
**FATE**	https://github.com/FederatedAI/FATE	Python-based Federated Learning platform with UI	Yes	Application	HBC	DP/MPC/HE	No	Yes	Yes
**NVFlare**	https://github.com/NVIDIA/NVFlare	Python-based Federated machine learning library	Yes	Library	HBC	None	No	No	Yes
**APPFL**	https://github.com/APPFL/APPFL	Python-based Federated machine learning library	Yes	Library	HBC	None	No	No	Yes
**COLLAGENE**	https://github.com/harmancilab/COLLAGENE	C++ SEAL wrapper with threshold multiparty keys	Yes	Library	HBC	HE/MPC	Yes	Yes	Yes

Libraries such as FedML [43] enable users to build machine learning applications with a specific focus on deep learning methods. Of note, PySyft [44, 45] integrates MPC (e.g., SPDZ scheme [75, 76] that requires a central key and share generator [48]) and data encryption to protect the shared intermediate gradient information for training machine learning models. However, there is a strong reliance on specific types of models that can be built using these libraries. In addition, the security of intermediate statistics is not easily modified as they are integrated into the source code. Recent interest in federated machine learning model training led to development of newer libraries [77–81], predominantly by industry efforts [80], with varying degrees of user-friendly interfaces (Flower [79]) to more low-level control on parameters (OpenFL [77], Vantage6 [78]). These libraries provide varying levels of privacy protection for the summary-level data and mainly rely on protection provided by aggregated statistics. Notably, some libraries use modified programming languages (e.g., SEQURE [61]), which can help optimize low-level implementations at the expense of complexity and maintainability.

Among the library implementations, Palisade and Lattigo are implementations of several lattice-based homomorphic encryption schemes to enable secure data analysis. These libraries also include the implementations of key sharing approaches (e.g., Asharov’s approach) for building collaborative tools. Usage of Lattigo and Palisade may be hindered by the necessity to implement the functions from scratch, and knowledge of details of parameter selections in HE. In comparison, COLLAGENE aims to provide more application-level functionality and ease of deployment specifically for collaborative scenarios. COLLAGENE also provides default file network I/O options and removes the necessity to implement the network functionalities by the users. Similar libraries, such as SEAL, provide single-key HE functionalities that are useful for building outsourcing services but SEAL currently does not have implementations for collaborative data analysis. TenSEAL [82] is a python-based wrapper for the SEAL library and provides tensor-level operations for building machine learning applications. TenSEAL’s current implementation does not provide an explicit interface for building federated tools.

There are also more focused library development efforts from industry and academia, e.g., Intel HE-toolkit [83], and PyFHEL [84], for utilizing HE-based operations. These are generally at the programming interface level and focus on single-key encryption without the network interfaces and collective decryption functionalities. In addition, GenoPPML [85] is a recently developed framework that combines MPC-based primitives with DP specifically for privacy-aware training regression models that use genomic data. In comparison to these, COLLAGENE aims to be more application-oriented with a focus on providing functionality at the command line interface level to make it more seamless to build collaborative methods.

### Federated genome-wide association testing (GWAS) and meta-analysis for binary traits

For demonstrating COLLAGENE’s usage, we implemented a federated binary-trait GWAS protocol using the matrix-level arithmetic operations provided by COLLAGENE. For binary-trait GWAS, we implement a federated logistic regression to perform a variant-level scoring test. Our implementation adopts the score test approach of the highly efficient GMMAT [70] algorithm that separates GWAS into two distinct steps. GMMAT relies mainly on matrix algebra and is amenable to a secure conversion by the COLLAGENE suite of tools. In essence, GWAS for binary traits relies on the relationship between genotype, covariates, and the phenotypes that are formulated by a generalized linear model:

$$E\left({Y}_{i}\right)={g}^{-1}\left({X}_{i}\alpha +{G}_{ij}{\beta }_{j}\right)$$

where $Y$ is an $N\times 1$ vector of phenotypes of $N$ individuals, $X$ is an $N\times p$ matrix of the $p$ covariates (including an intercept term), and $G$ is the $N\times M$ genotypes vector for $M$ variants. ${\alpha }_{p\times 1}$ and $\beta$ are the covariate and genotype weights, respectively. Finally, $g\left(\cdot \right)$ denotes the link function that links the relationship between the expected value of the phenotypes and the linear combination of the predictors. For binary traits, the logit link is used for quantifying the log-odds ratio of the case-vs-control subjects. GMMAT solves the generalized linear model by first fitting a null model that does not use genotypes:

$$E\left({Y}_{i}\right)={g}^{-1}\left({X}_{i}{\alpha }_{0}\right)$$

where ${\alpha }_{0}$ denotes the null model weights that do not rely on the genotypes. After null model fitting, GMMAT estimates the score test statistic and its variance using the genotypes and the null model predicted phenotype values. The separation of these steps not only makes GWAS efficient but also simplifies the privacy considerations in federated implementation:

1. Null model fitting (Fig. 3a). This step fits the null model weights, i.e., ${\alpha }_{0}$, using only the covariates in the model via a federated iteratively re-weighted least squares (IRLS) approach (Methods). Notably, this step does not make use of the sensitive genotype data from any of the sites. The non-reliance on sensitive genotype data alleviates the main genomic privacy concerns. Our current implementation decrypts the intermediate estimates of the null model parameters. This is deemed safe since the null model weights are usually very small in dimension (At most 10–20 covariates per study) compared to the aggregated sample sizes (tens of thousands) and do not leak much-identifying information. No other individual-level or summary-level information is shared among sites in cleartext form. Null model fitting is performed in 8 steps that first calculate a weighted covariance matrix of the covariates and invert it (Steps 1–5). Next, the null model parameter estimates are updated to be used in the next iteration of model fitting (Steps 5–8) (Methods).

**Fig. 3:**
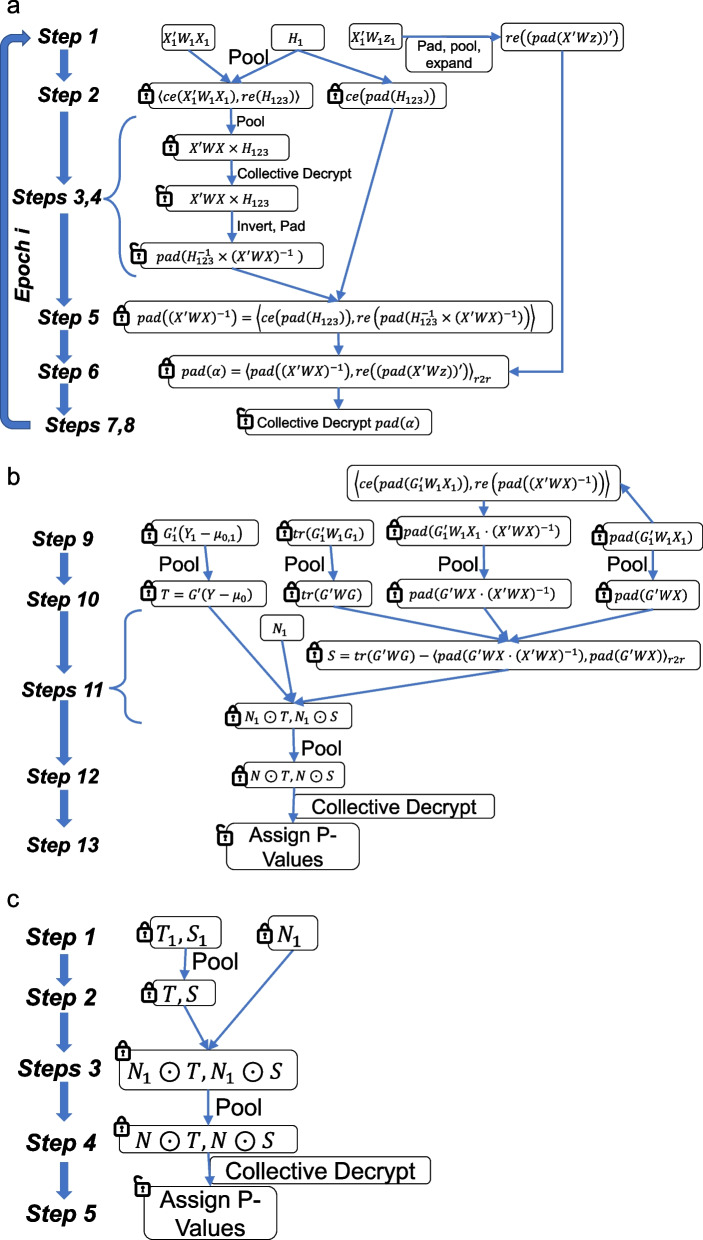
Illustration of federated GWAS algorithm. **a** 8 steps of null model fitting that is used in the GWAS protocol. First 4 steps utilize the matrix inversion (Fig. 2d) using the mask matrices ${H}_{1}$, ${H}_{2}$, and ${H}_{3}$ to calculate the encrypted inverse of the pooled covariance matrix of covariates, i.e., ${\left({X}^{\prime}WX\right)}^{-1}$. This matrix is also padded to the next power of 2 for usage later. In step 6, the weights are updated using a row-row multiplication, i.e., ${\alpha =\left({X}^{\prime}WX\right)}^{-1}\cdot \left({X}^{\prime}Wz\right)$. The parameter estimates for the current epoch is collectively decrypted and used in the next iteration. **b** 5 steps of *p*-value assignment, denoted by steps 9–13. Each site first calculates the components of the *p*-value statistics using local genotype and phenotype data. These are, $T={G}_{1}^{\prime}\left({Y}_{1}-{\mu }_{\mathrm{0,1}}\right)$, ${G}_{1}^{\prime}{W}_{1}{G}_{1}$, ${G}_{1}^{\prime}{W}_{1}{X}_{1}\cdot {\left({X}^{\prime}WX\right)}^{-1}$, and $G^{\prime}WX$. Next, each matrix is pooled among sites (Step 10) and the scale parameter is calculated, i.e., $S={tr(G}^{\prime}WG)-{\langle {G}^{\prime}WX\cdot {\left({X}^{\prime}WX\right)}^{-1},{G}^{\prime}WX\rangle }_{r2r}$, where $tr(A)$ denotes the trace of matrix $A$. Next, each site generates a mask vector, denoted by ${N}_{1}$, and elementwise multiplies with both $T$ and $S$ vector with the same mask vector. The masked statistics are pooled among sites to calculate the final collectively masked statistics, which are collectively decrypted and used for assigning final *p*-values. **c** The meta-analysis steps. These steps start from the $S$ and $T$ statistics that GMMAT calculates. Each site performs the masking, pooling, and collective decryption followed by the *p*-value assignment step
2. Scoring of the variants using the null model (Fig. 3b). After null model fitting, each variant is scored using the score test of GMMAT. This step protects the genotype data and related summary-level matrices. It relies on first calculating the encrypted score statistic and its variance (Steps 9, 10), followed by secure collective decryption of the statistics (Steps 11–13) (“Methods” section).
3. Secure meta-analysis (Fig. 3c). The meta-analysis relies on the pooling of the score test statistic and the variance estimate from different sites. This is performed in 5 steps as shown in Fig. 3c. Meta-analysis is highly efficient as the only necessary operation is the summation of the statistics from all sites. Similar to steps 11–13 of Fig. 3b, we mask the score and scale parameters using pooled site-specific mask vectors, which are then pooled and collectively decrypted. The final results conserve the normalized score test statistic (which is distributed as per chi-squared distribution with 1 degree of freedom) that is used for *p*-value assignment.

### Comparison of secure federated GWAS with plink2

**Fig. 4:**
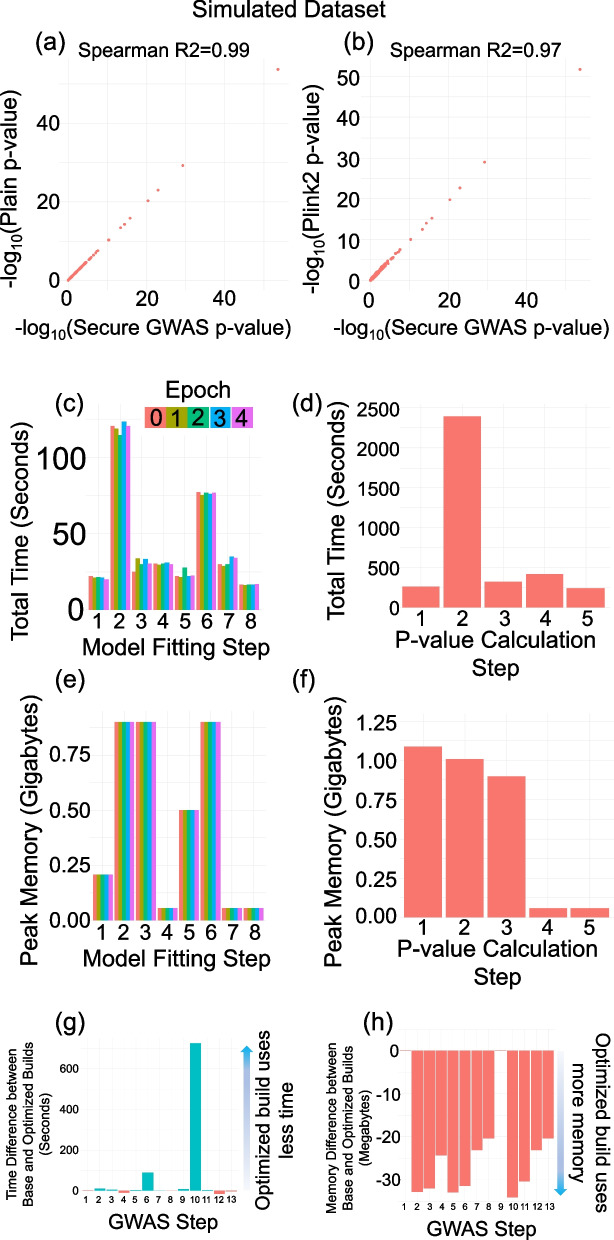
GWAS *p*-value concordance and time/memory requirements. **a** The *p*-value concordance between secure federated GWAS (*x*-axis) and plain federated GWAS (*y*-axis) using the simulated genotype data among 3 sites. The Spearman *R*^2^ of the correlation is reported at the top of each plot. **b** The *p*-value concordance between federated GWAS (*x*-axis) and plink2 GWAS using pooled simulated dataset (*y*-axis). **c** The time usage of secure federated GWAS (in seconds) at each step of null model training (*x*-axis) and at each epoch (bar colors). **d** The time usage of secure GWAS for *p*-value assignment. **e** Peak memory usage (in gigabytes) of secure null model fitting. **f** Peak memory usage of secure *p*-value assignment. **g** Difference between total time usage (in seconds) of GWAS protocol using base and optimized build of COLLAGENE. Each bar shows the total time usage difference between base and optimized builds for one step in the protocol. For steps 1–8 (null model fitting with multiple epochs), time usage of the protocol is summed over all epochs. Blue (red) bars indicate that optimized build requires less (more) time than base build. **h** Difference between peak memory usage (in megabytes) for base and optimized build of COLLAGENE. Each bar shows the difference in memory usage for one step in the protocol. Red bars indicate that optimized build uses slightly more memory

We compared the secure federated GWAS testing approach with plink2 using simulated and real datasets. In simulations, we generated datasets by simulating population-specific genotype data for 3 sites where sites harbored European, East Asian, and African genomes, respectively (Methods). The genotype data at each site was set to 4800 subjects that contained the genotype values (0,1,2) for 57,344 variants (number of variants that fit 7 ciphertexts). Gender was randomly assigned to each subject with 50% male/female probability. Eight population-level covariates were estimated by projection of the study subject genotype onto the 3 reference populations (European, East Asian, and African) from the 1000 Genomes panel. The binary phenotypes were simulated using a logit link linear model whose weights were randomly selected including a gender-specific fixed effect (Methods). We ran plaintext (unprotected) federated GWAS, secure federated GWAS, and plink2-based pooled GWAS using the genotype–phenotype dataset from all sites. We observed high concordance between secure and plaintext-federated GWAS results (Spearman *R*^2^ between *p*-values assigned by the methods was higher than 0.99, Fig. 4a), which indicates that secure protocol accurately replicates the expected results. We also observed high concordance to plink2 results for which the correlation between *p*-values was 0.97 (Fig. 4b).

These results highlight that COLLAGENE’s toolbase can be used for accurately implementing a real use case and demonstrates the potential of COLLAGENE for new tool development.

#### Time and memory requirements

We next evaluated the time and memory requirements of federated secure GWAS as implemented by COLLAGENE’s toolbase. We used the simulated sample set (14,400 samples divided among 3 sites). The sites used an AWS S3 bucket to share the encrypted intermediate matrices. We modified our testing scenario to use 6 covariates to be more compatible with the testing scenario that was used in sPLINK, another privacy-aware federated method that utilizes the HyFED framework, which requires 2 trusted entities (Aggregator and Compensator) that participate in the protocol for removing the noise in global model parameters. This framework is comparable to our secure GWAS setting as both frameworks are privacy-aware and federated.

From a security perspective, Aggregator and Compensator entities in sPLINK take part in sensitive data processing and there can be risks around sensitive leakage with the existence of curious colluding entities. Although this is unlikely, the main issue for the usage of sPLINK is how well the usage of Aggregator/Compensator sites can be justified in the presence of the regulatory requirements. Our approach, however, relies only on the *KeyMaker* who does not take any part in the sensitive data processing and the collusions among collaborating sites should not lead to data leakage.

We focused on 57,344 variants and measured the throughput of single-threaded secure GWAS. The whole calculation is finished in 5346 s (approximately 1.49 h) (Fig. 4c, d) with peak main memory usage of 1.09 GB (Fig. 4e, f). Compared to the sPLINK, which processed approximately 58,000 variants per hour per thread (estimated from the result as reported in Figure 7a in Nasirigerdeh et al. [74], “Methods”), which indicates that our implementation has slightly lower throughput. This is expected since our implementation does not make use of the Aggregator/Compensator entities to remove noise from intermediate datasets and relies on HE to protect intermediate statistics (except null model weights) with much lower potential risks as a results of collusion among curious entities.

As our approach separates the null model fitting from the *p*-value assignment, we next separated the total time required for null model fitting and *p*-value assignment steps. The complete protocol finished in 5346 s in which model fitting and *p*-value assignment were completed in 1761s (0.48 h) and 3585 s (0.99 h), respectively. This indicates that after the model fitting step, each thread processes 57,344 variants per hour (i.e., 57,344 variants are processed in 0.99 h). This is an important quantity since the model fitting is required only once and sites can re-utilize the model to perform *p*-value assignment in larger variant sets without the additional requirement of model fitting.

In terms of network traffic, each site used 1.49 GB of network traffic over a total of 490 network requests, which totals 4.5 GB of network traffic over all sites. Our network usage is higher compared to sPLINK’s usage of 1.6 GB (As reported in Figure 7b in [74]). This difference can be partially explained by the fact that secure protocol transfers only encrypted data which are inflated in size by the ciphertext expansion rate (i.e., size of encrypted matrix divided by size of plaintext matrix), which is approximately 22.1 (“Methods”). There are, however, other factors that may impact differences. For example, sPLINK and our protocol use fundamentally different architectures (sPLINK uses central aggregator/compensator while our protocol relies on encrypted data exchanges from shared disk space) and methodology such as differences in model optimization (Newton-Raphson in sPLINK and IRLS in secure protocol) and estimation of *p*-values. The difference in network traffic is therefore a result of these factors. Furthermore, our approach separates the null model fitting from *p*-value assignment and exchanges only null model parameters in the model fitting stage (i.e., 1 ciphertext per site per exchange, which is around 1 MB in size). This stage can be optimized to make full utilization of the ciphertexts by concatenating several matrices together. In the *p*-value assignment stage (Table 2), the ciphertexts are fully utilized to ensure no slot space is wasted in the exchanges.

**Table 2:** Total disk space storage and network usage by each client. The two columns show disk storage and network transfer size. Rows indicate the base and optimized build of SEAL that COLLAGENE was compiled with

	**Total disk storage**	**Total network upload/download**
**Base SEAL**	4.18 Gb	1.48 Gb
**Optimized SEAL**	3.60 Gb	1.19 Gb

We next assessed how much of the computation time is taken by network traffic. We ran our secure GWAS locally, i.e., all sites are simulated to share the same file system without any network exchanges. The overall protocol finished in 3092 s (0.86 h) wherein the model fitting required 877.8 s and the *p*-value assignment was completed in 2214 s. When compared with networked protocol that finished in 5346 s, this indicates that network transfers required 2254 s (5346 minus 3092), approximately 42% of the computation time (2254 s out network of 5346 s of total time) for federated GWAS (A similar result was reported by sPLINK.) Overall, network traffic is a major bottleneck for collaborative studies and is an important factor in designing federated protocols that have a lower network footprint for increasing efficiency.

#### Software and hardware-level optimization of storage and ciphertext processing

In the above experiments, COLLAGENE was compiled with a baseline build of the SEAL library that does not make use of the ciphertext compression (removes redundancy in the serialized ciphertext file format) for decreasing storage and the AVX512-enabled optimizations using that enable streamlined ciphertext processing using Homomorphic Encryption eXtensions Library (HEXL) [86, 87]. While these optimizations are partially CPU-specific, new Intel and AMD family processors support the AVX512 instruction set. We re-compiled SEAL and COLLAGENE’s matrix library with these operations and measured the run time with these optimizations to evaluate which steps benefit from these optimizations. Also, while these optimizations do not reflect innovations from COLLAGENE’s design, they provide a baseline for the best performance that COLLAGENE can deliver.

The federated calculation completed the GWAS pipeline in 4547 s (1.26 h) with an optimized build of COLLAGENE. Of this total time, 1652 s (0.46 h) were spent for null model training, and 2895 s were spent (0.80 h) for *p*-value assignment (Fig. 4g). In comparison with the baseline build, we observed the main difference is at Step-6 where null model parameters were updated (Fig. 3a). This is expected as this step contains the most demanding HE operations (row-row matrix multiplications) that are optimized by the HEXL library. Similarly, the largest improvement for *p*-value assignment runtime was at Step-10 (Fig. 3b) where the sites pool the intermediate encrypted matrices (Fig. 4g). Peak memory usage of an optimized build of COLLAGENE was 1.1 GB slightly higher (around 30 MB) than the baseline build (Fig. 4h). We also observed that the network I/O and storage were decreased in comparison to the baseline build. The optimized build of COLLAGENE used around 1.2 GB of network I/O and in total 3.6 GB were used for storage at each client (Table 2). In summary, the optimizations in the underlying SEAL library make observable improvements in the performance.

#### Comparisons with real data

We next compared the secure federated GWAS pipeline with plink2 using the late-onset Alzheimer’s disease (LOAD) Cohort that comprises 2545 subjects that were accessed through the database of genotypes and phenotypes (dbGaP) study accession identifier phs000168.v2.p2. For simulating federation, we shuffled and horizontally split the dataset among 3 sites with a similar number of subjects (848 subjects per site, 6 population covariates, and 1 gender). The genotype data comprised 557,056 variants. For decreasing the network cost associated with AWS transfers, we used a directory on local file system as the shared disk space rather than a shared AWS bucket (no changes in the underlying encrypted protocol).

**Fig. 5:**
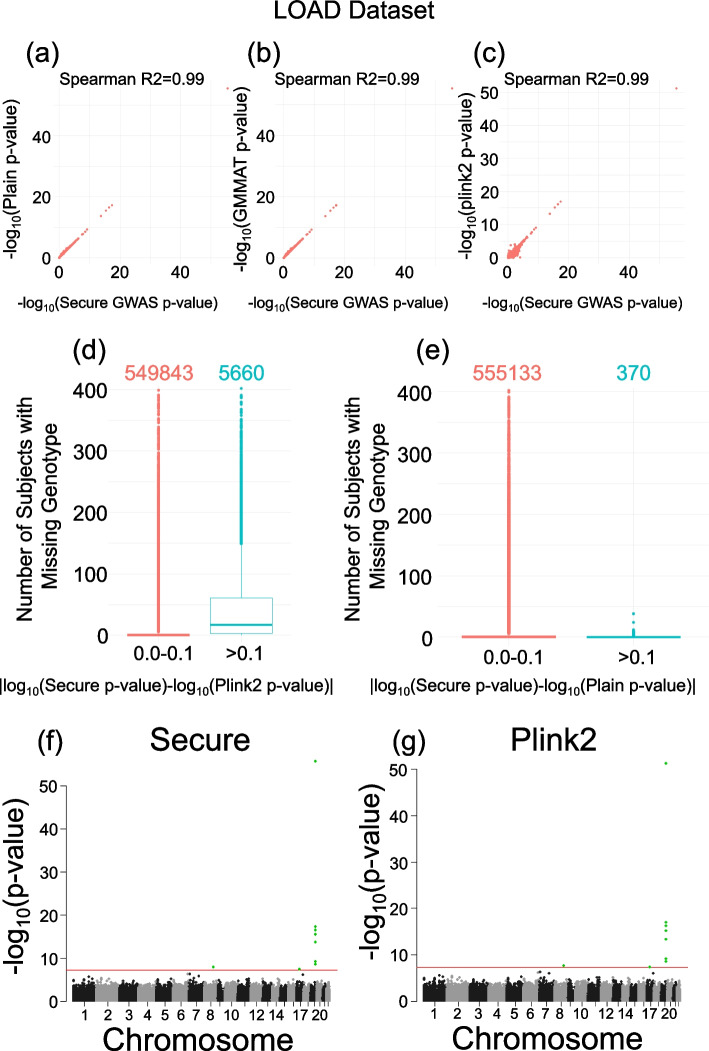
GWAS results using LOAD dataset with secure and plain protocols, GMMAT, and plink2. **a***P*-value concordance between secure and plain federated GWAS using LOAD dataset. **b** Concordance between secure protocol and GMMAT. **c** Concordance between secure protocol and plink2. **d** The distribution of number of missing subjects per variant (*y*-axis) stratified by difference in absolute log_10_(*p*-value) assigned by secure protocol and plink2. The bars correspond to low (red) and high (blue) *p*-value difference. Number of variants is shown at the top of the bars. **e** The distribution of missing subjects per variant stratified by difference in absolute log_10_(*p*-value) assigned by secure and plain federated protocol. The bars correspond to low (red) and high (blue) *p*-value difference. **f** Manhattan plot shows the variant position (*x*-axis) vs − log_10_(*p*-value) for secure protocol. Red horizontal line shows 5 × 10^−8^ cutoff for the *p*-value. **g** Manhattan plot shows the variant position (*x*-axis) vs − log_10_(*p*-value) for plink2

We executed the COLLAGENE-based secure and plaintext-federated protocols, and plink2 on the pooled dataset. We also ran GMMAT on the pooled dataset to have a separate baseline while comparing methods. We first compared the *p*-values assigned to the variants by each method. The *p*-values assigned by plaintext-federated protocol and GMMAT are concordant to secure protocol (Fig. 5a, b, Spearman *R*^2^ 0.99), which indicates that secure protocol replicates the federated plaintext result and GMMAT’s pooled analysis results with high concordance. Similar comparison between plink2 and secure protocol showed a high concordance between the methods as well (Fig. 5c, Spearman *R*^2^ = 0.99).

We focused on the variants that exhibit the largest differences between plink2 and secure federated protocol. Of note, we do not expect a perfect concordance because the score test has different statistical properties than plink2’s approach (e.g., score test asymptotically converges to the chi-squared distribution under null hypothesis). We focused on the variant allele frequency and missingness as the possible source for differences. Concordant with GMMAT protocol, the secure protocol assigned mean imputed genotypes to each variant while plink2 excludes, for each SNP, the subjects with missing genotype. We observed that the SNPs with high *p*-value difference exhibit high missingness (Fig. 5d, e). This result shows that missingness may create minor differences between methods. We did not observe correlation between *p*-value differences (of secure protocol and plink2) versus variant allele frequencies.

We next evaluated the top SNPs identified as significantly associated with phenotype (i.e., AD diagnosis). Manhattan plots (Fig. 5f, g) show that the most highly associated SNPs are located on 19q13.3 [88], which is a known locus that is associated with AD status. We extracted the SNPs that pass the GWAS threshold (5 × 10^−8^) and found 9 SNPs that are identified by secure protocol, GMMAT, and plink2 (Table 3). Overall, GMMAT and secure protocol assigned the same SNPs to be statistically significant. Among these variants, 8 of them were marked as significant by plink2. The remaining variant, rs4796606 on chr17:36917613 (hg18), was assigned borderline *p*-value (5.38 × 10^−8^) by plink2.

**Table 3:** 9 SNPs that were significant with respect to GWAS cutoff 5 × 10^−8^. ^a^rs4796606 is identified by 3 methods

**Variant**	**Position**	**Assigned *P*-value**				**Alternate allele frequency**	**Missing subjects**
		**Plink2**	**Secure federated**	**Plain federated**	**GMMAT**		
rs2075650	19:50087459	6.43E-52	2.72E-56	2.64E-56	3.96E-56	0.2766	1
rs6859	19:50073874	1.21E-17	5.37E-18	5.21E-18	5.88E-18	0.5102	0
rs405509	19:50100676	6.93E-17	3.55E-17	3.52E-17	3.95E-17	0.4393	0
rs8106922	19:50093506	7.31E-16	3.52E-16	3.49E-16	3.88E-16	0.3081	1
rs157580	19:50087106	4.48E-14	1.94E-14	1.90E-14	2.09E-14	0.702	4
rs439401	19:50106291	7.92E-10	6.27E-10	5.94E-10	6.33E-10	0.7016	0
rs10402271	19:50021054	3.34E-09	2.73E-09	2.71E-09	2.87E-09	0.3764	0
rs3103765	8:96460015	2.26E-08	1.45E-08	1.43E-08	1.51E-08	0.5122	0
**rs4796606**^**a**^	17:36917613	5.38E-08	3.40E-08	2.69E-08	2.83E-08	0.9387	0

### Comparison of meta-analysis of association testing results

Meta-analysis of GWAS summary statistics is a computationally efficient approach for combining GWAS results from multiple sites. We adopt the meta-analysis strategy of the GMMAT tool that combines the appropriate statistics from individual sites. However, sharing the SNP-level summary statistics can create privacy concerns. We utilize COLLAGENE’s matrix masking procedure that combines noise from all sites and hides the summary statistics that are being aggregated in the meta-analysis while preserving the final meta-analysis result (Steps 11–13 in Fig. 3b). The final results that are collectively decrypted by all sites can only be used for estimating the significance of variant association from the meta-analysis.

**Fig. 6:**
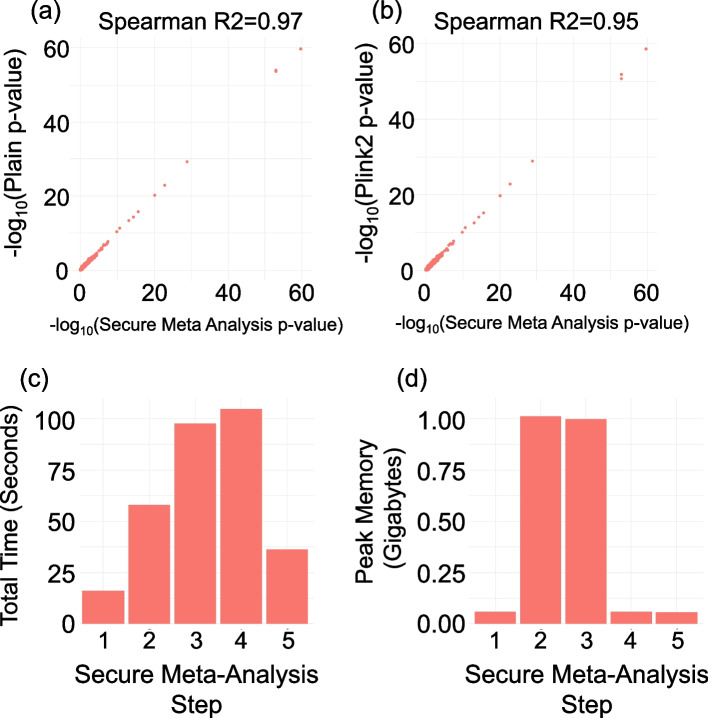
*P*-value concordance and resource requirement of secure meta-analysis. *P*-value concordance of secure meta-analysis *p*-values with **a** plaintext protocol and **b** plink2. **c**, **d** Time/memory requirements of secure meta-analysis

To test meta-analysis, we executed the secure meta-analysis protocol among 3 sites using the data from our population-specific site dataset used in previous comparisons. Here, each site first locally executed GMMAT on the local genotype/covariate/phenotype dataset. Next, the score test statistics are extracted and encrypted. The meta-analysis protocol is executed using an S3 bucket for storing the encrypted intermediate data files (the encrypted masked summary statistics) using a single thread per site. Compared to the *p*-values assigned by plink2 on the pooled sample set, the secure meta-analysis results were highly concordant (Fig. 6a, b) when compared in terms of rank-based correlation of *p*-values. We, however, did observe a slight decrease in the concordance of *p*-values between the secure meta-analysis and plink2 (Spearman *R*^2^ statistic for plink2-vs-meta-analysis in Fig. 6b is 0.95) when compared with the concordance of *p*-values in federated GWAS analysis (Spearman *R*^2^ for plink2-vs-Federated GWAS is 0.97 Fig. 4b). This is expected because meta-analysis uses summary statistics while federated GWAS uses raw data. Secure meta-analysis protocol finished in approximately 311 s (5.2 min) (Fig. 6c), indicating high efficiency in time usage. The peak main memory usage was approximately 1 GB (Fig. 6d).

In summary, our results indicate that COLLAGENE’s toolbase can be used in a real-world scenario for building federated and secure data analysis methods.

## Discussion

We presented COLLAGENE, a new framework for building collaborative and federated pipelines for the analysis of sensitive datasets. COLLAGENE combines HE primitives and multiparty calculations for decreasing development time and efforts. COLLAGENE encapsulates and relieves much of the underlying complexity around key sharing using *KeyMaker* service and HE-level implementations by providing command line tools and programming libraries that can be integrated into new data analysis pipelines. Primary emphasis of COLLAGENE is on enabling secure matrix operations, aligning with the widely used data representations in bioinformatics. Unlike numerous federated methods, it eliminates the need for a central instance that handles sensitive data because *KeyMaker* never receives sensitive data.

We advocate the usage of federated approaches that are supplemented using HE, MPC, and DP appropriately. COLLAGENE provides several options to implement these in an integrated fashion for matrix-formatted datasets, which encompasses most tools in the field of biomedical informatics and bioinformatics. Compared to the outsourcing-based approaches (e.g., TrustGWAS [89]) where the pooled and encrypted raw data is sent to an outsourcing site, the secure federated approaches are more promising in the efficiency of disk and network usage without sacrificing accuracy. In the setting of cloud computations where most outsourcing is currently performed, this directly impacts cost estimates: While storage costs may be manageable, the network costs (e.g., downloading data out of AWS S3 buckets) are charged once per transfer and they may become a strict bottleneck in the long term. This, however, requires the appropriate formulation and implementation of the protocols to ensure that algorithms are efficiently ported into the secure domain.

Several rules follow to ensure that risks are minimized against the assumed adversarial model that COLLAGENE targets. COLLAGENE assumes that collaborating sites are honest-but-curious entities [90], who adhere to the analysis protocols without malicious deviations. We believe that the majority of the adversarial entities in the biomedical research community can be considered as honest-but-curious entities [90] who are not actively trying to break protocols to steal individual-level information. Overall, when the protocols are appropriately designed, COLLAGENE should be effective for protecting against *accidental leakages* [91, 92] wherein a researcher may accidentally re-identify a study participant, e.g., linking two datasets may pinpoint an individual’s unique identifying information in a third dataset [93, 94]. The most prevalent privacy concerns in genomics and biomedical literature stem from re-identification concerns such as the scenarios laid out by Gymrek et al. [95] (linking genomics to genealogical datasets), Homer et al.’s *t*-test [38] and Sankararaman et al.’s likelihood ratio [96] test, and Bustamante et al.’s beacon attack [97], which are relatively easy to execute when large-scale summary statistics are available. Due to their simplicity, these attacks can be applied by honest-but-curious entities, without actively breaking protocols. We provide several suggestions against these attacks.

Most importantly, the sites must ensure that individual-level data is never shared with other sites even if it is encrypted. Any intermediate data that will be collectively decrypted should be aggregated to small dimensions with non-linear functions and be masked using strong masks (DP can set the noise levels) to thwart unmasking attacks [98–100]. The partial decryptions should be encrypted using symmetric keys that *KeyMaker* generates to avoid unauthorized accession. This is important since there are known attacks even on the encrypted data for revealing model parameters [101, 102]. To counter these attacks, COLLAGENE uses a fixed smudging noise level to sanitize partially decrypted data, which is set to 40 bits by default. The estimation of this “smudging” noise for ciphertext protection is an open problem and is an active field of research [103, 104].

We would like to note that COLLAGENE does not protect against malicious entities, which actively seek to deviate from protocols to steal data or disrupt the integrity and accuracy of the analysis. These entities may selectively target certain datasets (e.g., marginalized populations, stigmatizing conditions). Protection against these entities is computationally challenging [105]. Finally, regardless of whether the entities are considered malicious or honest-but-curious, sites must establish policies about usage of final results because these will be revealed to all collaborating sites. The privacy concerns around usage of final results should be accounted for based on the data access and usage policies among the participants for ensuring that the collaborating entities do not use the data for any unintended purposes.

There are several limitations of COLLAGENE that warrant further research. For example, it is necessary to put complete trust in the keys generated by the *KeyMaker* service. This is a fundamental issue faced by many practically feasible federated analysis frameworks because key generation is an integral step that relies on collective trust among parties. Any collusion among the parties can lead to partial leakage of keys. In our framework, we pool the trust on a single site, *KeyMaker*, which can be operated by a recognized entity (such as NIH or local regulators). In addition, *KeyMaker* does not (and should not) take part in the data analysis steps, i.e., even if *KeyMaker* starts acting maliciously, the sites can make sure that *KeyMaker* does not receive any data encrypted by the keys that it generated.

COLLAGENE currently lacks the key-switching and bootstrapping capabilities that single-user libraries have. We would like to note that these operations can be simulated using matrix masking. For example, the encrypted data can be masked, collectively decrypted, and re-encrypted in the target public key, and finally, the mask can be removed. Similarly, the bootstrapping can be simulated by a ciphertext-refreshing operation: Sites can first add a collective mask to the encrypted data matrix, collectively decrypt it, re-encrypt it, and finally remove the noise to generate a freshly encrypted data matrix. It should be noted that currently available implementations of bootstrapping require careful parameter selection (e.g., TFHE, Lattigo) that makes it challenging for practical use in real-life scenarios, especially for non-experts. For advanced users, these implementations can serve as more useful options. We are currently implementing key-switching operations directly on the ciphertext-level data and exploring the user-friendly implementation of bootstrapping to integrate these functionalities into COLLAGENE. COLLAGENE’s current matrix arithmetic library delivers baseline performance but the performance can be improved using more convenient methods. We are planning on integrating more matrix encoding techniques and expanding the toolbase to increase the efficiency of some of the operations. This, however, usually incurs a cost on disk and network storage in the collaborative analysis settings. We leave these considerations to the future development of COLLAGENE.

Another limitation of COLLAGENE is the privacy concerns around sharing of the unprotected null model parameters in the federated binary phenotype GWAS. Although this is a case-specific limitation, it is important for users while building new secure pipelines. As we described above, the currently implemented model shares the null model parameters in plaintext (unprotected) among sites. Because the null model parameters are a certain type of summary statistic, sharing them may cause concerns around re-identification attacks. This is, however, rather unlikely for several reasons. First, the null model parameters are calculated once using only a small number of covariate information compared to the large sample sizes. Covariates are, unlike genotypes, not expected to leak much individual re-identifying information since they are a small number of nuisance parameters that are used for correcting the fixed population stratification and gender effects. Notably, including too many known and non-confounding covariates in a GWAS study (or any analysis tool) can also hinder the power of the study as the null model becomes too conservative [106].

Secondly, the null model parameters are highly aggregated and non-linear functions of the covariate information from all sites. The contribution of each individual to the null model parameters is in turn a complex function of the covariates. It should also be noted that IRLS, by design, rejects outlier data points by automatically down-weighting them in the parameter inference [107]. This property of IRLS is advantageous from a privacy-preserving point of view since the contribution of outlier samples is down-weighted in the final parameter estimates. Since the accuracy of the statistical re-identification attacks that make use of high dimensional summary statistics (e.g., variant-level summary statistics in Homer et al. [38]) relies on a direct relationship between the individual-level data and the summary statistic, it is unlikely that knowledge of the covariates for one individual will be sufficient to evaluate their participation into the study [108]. We would like to re-iterate that this is a case-specific limitation that is not inherent to COLLAGENE and leave the analysis of identifying information leakage from the null (covariate-only) model parameters as a future research direction.

## Conclusions

COLLAGENE is a novel framework that can accelerate development of collaborative pipelines. COLLAGENE aims to make secure and collaborative genomic data analysis more easily accessible. It can be flexibly expanded into more focused development efforts or it can be used for simply encrypt-aggregate-decrypt operations for securing intermediate data [109]. Its tool base can be expanded with community-driven efforts such as iDASH genomic privacy challenges [110].

## Methods

We present the details of the algorithms underlying the COLLAGENE framework.

### Considerations around encryption parameter selection

COLLAGENE uses CKKS as the default homomorphic encryption scheme [111, 112] with the underlying primitives implemented by the SEAL C++ library [65]. The encryption parameters are set using a configuration file, which stores the number of bit sizes for the residual number system (RNS) representation of the ciphertext coefficient modulus (referred to as $\mathrm{log}\left(q\right)$), the polynomial degree modulus ($n$) for describing the CKKS scheme. In general, the length of the modulus bit sizes vector is equal to the multiplicative depth of the evaluation using the keys with these parameters. The polynomial degree modulus relates to the slot size, i.e., the number of plaintext numbers one can store in each ciphertext: The slot size is exactly half of the polynomial degree modulus. The final parameter is the bit size for noise that is added to the secret key shares generated by *KeyMaker*, which is only necessary for *KeyMaker*. COLLAGENE provides options to ensure that the selected $n$ and $\mathrm{log}\left(q\right)$ parameters adhere to the required security levels.

COLLAGENE provides several options for making it easier for users to interpret and set these parameters in accordance to the protocol that will be executed among sites. The length of the coefficient modulus bit sizes vector determines how many multiplications the protocol can perform before decryption fails. For algorithms that require a large number of multiplications (i.e., deep neural networks or function approximations with large polynomial degrees), it is necessary to use longer bit size vectors. This, however, degrades security (requires larger $n$) and also the performance as each operation needs to loop over all the decomposition levels in the bit size vector. To get around this limitation, users can perform masked matrix re-encryption (i.e., ciphertext refreshing) on “exhausted” ciphertexts using COLLAGENE and use the freshly encrypted ciphertext to keep operating on the data. The options that should be used eventually depend on the multiplicative depth of the algorithm and we recommend users to consider formulation of the algorithms to decrease multiplicative depth of calculations.

### Ciphertext expansion rates

Ciphertext expansion refers to inflation of size of ciphertext data compared to the underlying plaintext data. To estimate the ciphertext expansion rate for the GWAS protocol, we generated a random 64 × 128 matrix (full utilization of a ciphertext that can hold 8192 entries) and encrypted it using COLLAGENE, and calculated the expansion rate as

$$expansion\, rate=\frac{size\, of\, encrypted\, matrix}{size\, of\, plaintext\, matrix }.$$

We repeated this estimation 100 times and found that the mean expansion rate is 22.1 with standard deviation of 0.08.

### Secret key sharing protocol by *KeyMaker*

Before the generation of keys, the sites agree on the calculation that will be performed (e.g., collaborative GWAS) and have the a priori knowledge of multiplicative depth necessary for executing the calculations (bit size vector of coefficient modulus). The sites agree on the encryption parameters for the calculation. Each site generates a public/private key pair that will be used for encrypting/decrypting their distributed CKKS secret key share (DSK) generated by *KeyMaker*. We refer to these first set of keys as DSK encryption keys. Each site sends their DSK encryption key to one of the sites that initiates the key generation (Fig. 1). The key sharing used by *KeyMaker* is implemented by modifying SEAL’s secret key generation routine.

#### Generation of DSKs

Given $S$ sites whose DSK encryption keys are sent to *KeyMaker*, *KeyMaker* first generates one master CKKS secret key, $msk$, which is a vector of length $n$ whose elements are selected from $\left\{-\mathrm{1,0},1\right\}mod\left(q\right)$. Next, *KeyMaker* generates a noise vector for each site that will be added to $msk$ to generate the DSK for the corresponding site. The noise vector is also a length $n$ vector whose elements are selected from a discrete Gaussian noise with variance equal to the DSK noise level (${\sigma }_{DSK}^{2}\propto {2}^{{\lambda }_{dsk}}$) that is specified in the parameters. For each of the $p$ entries in the master key, *KeyMaker* samples the noise vector for all sites from the DSK noise levels. This process is done for all sites except one site, which receives the negative of the total DSK error:

1. At key $msk$ entry $j\le n$, shuffle the site indices $[1,S]$, and select a random site index in which the master key’s value will be added to. We denote this site index with ${s}^{*}$.
2. Select the next site index, ${s}_{next}$, among the shuffled indices
3. If $${s}_{next}={s}^{*}$$, set $${dsk}_{{s}_{next},j}=ms{k}_{j}$$
4. If this is not the last to be assigned, set its $dsk$ entry as: ${dsk}_{{s}_{next},j}\leftarrow {dsk}_{{s}_{next},j}+{e}_{DSK}, {e}_{DSK}\propto N\left(0, {\sigma }_{DSK}^{2}\right)$
5. If this is the last site to be assigned a key value, set it to ${dsk}_{{s}_{next},j}=\left({dsk}_{{s}_{next},j}-{\sum }_{s\ne {s}_{next}}ds{k}_{s,j}\right) mod \left(q\right)$

The basic idea in this approach is to ensure that $\forall j\le n, \left({{\sum }_{s}dsk}_{s,j}\right)mod\left(q\right)=ms{k}_{j}$. At each entry of the secret key, the master key is added exactly to one of the randomly selected sites and the complementary noise is added to all of the sites. This procedure requires very small computational resources and is performed in memory without any disk accession. The master secret key is discarded after key shares are generated. COLLAGENE implements the secret key sharing by accessing and modifying the internal arrays that store the secret key coefficients.

Next, *KeyMaker* uses the master secret key to generate a public key that will be shared with all sites:

$$\left({pk}_{0}, {pk}_{1}\right)=\left(-a\cdot msk+e, a\right) mod \left(q\right)$$

where error polynomial $e$ is sampled from a discrete Gaussian distribution and $a$ is sampled from uniform distribution. Note that the public key is composed of two polynomials ${pk}_{0}, {pk}_{1}$, which are the same length as the secret key. *KeyMaker* uses SEAL’s native interface to generate the public key. After all DSKs are collected, *KeyMaker* encrypts each site’s secret key share with the DSK encryption (public) key of the site. This way, key share for each site is protected from other sites and outside malicious entities. *KeyMaker* also creates a symmetric encryption key that is to be shared among all sites. This symmetric key is used for encryption/decryption of partially decrypted data in collective decryption protocols. *KeyMaker* also generates the relinearization keys, and the Galois keys, which are necessary for processing encrypted data. The final set of keys (encrypted DSKs, public, relinearization, and Galois keys, and common symmetric key encrypted by DSK encryption key) are stored in a tar archive file and returned to the sites for downloading. It should be noted that *KeyMaker* does not have any further role in the data analysis.

### Encryption and collective decryption protocol

Encryption of plaintext data is done using the conventional RLWE procedure as implemented in SEAL. COLLAGENE adds wrappers to simplify encryption and also implements the collective decryption operation.

#### Data encryption

Given a plaintext matrix $m$, the matrix is flattened and encoded using a CKKS encoder using SEAL (SEAL::CKKSEncoder). Next, the ciphertext polynomials are calculated: ${c}_{0}=\left(p{k}_{0}+m\right) mod q$, ${c}_{1}=p{k}_{1}$ where $m$ is the plaintext polynomial for the encoded plaintext matrix. This is encapsulated in encryption option of COLLAGENE, which is performed by SEAL library (SEAL::Encryptor). For encryption, COLLAGENE takes as input the common public key and the plaintext data matrix that will be encrypted and uses SEAL’s native encryption function. Of note, the polynomial multiplication (addition) operations in key generation and encryption correspond to polynomial modulus multiplication (addition). When needed, COLLAGENE uses SEAL’s optimized implementations for polynomial arithmetic.

#### Collective decryption

Given a ciphertext, $ct$, and the corresponding master secret key, decryption in SEAL is performed as:

$$dec\left(ct=\left({c}_{0},{c}_{1}\right);msk\right)=\left({c}_{0}+msk\cdot {c}_{1}\right) mod \left(q\right)$$

It can be shown from above equation that $dec\left(ct=\left({c}_{0},{c}_{1}\right);msk\right)=m+e{\prime}$, where $e{\prime}$ is the accumulated plaintext noise in the ciphertext. This decryption operation is implemented into SEAL::Decryptor class.

For the multiparty key scenario, no entity has access to the master secret key. We can, however, use the secret key shares in the collective decryption. We can write the decryption operation in terms of the secret key shares:

$$dec\left(ct=\left({c}_{0},{c}_{1}\right);msk\right)=\left({c}_{0}+msk\cdot {c}_{1}\right) mod \left(q\right)=\left({c}_{0}+\sum \limits_{s}\left(ds{k}_{s}\cdot {c}_{1}\right)\right) mod \left(q\right)={c}_{0}+\left(ds{k}_{1}\cdot {c}_{1}+ds{k}_{2}\cdot {c}_{1}+\dots +ds{k}_{S}\cdot {c}_{1}\right) mod \left(q\right).$$

It can be seen that each site can calculate a “partial decryption” (i.e., ${c}_{1}\cdot ds{k}_{s} mod\left(q\right)$) and share it with other sites. The final decryption can be calculated by aggregating the partial decryptions from all sites and adding ${c}_{0}$ polynomial once to obtain $dec\left(ct=\left({c}_{0},{c}_{1}\right);msk\right)$. The partial decryption at each site is:

$$pardec\left(ct;ds{k}_{s}\right)=\left({ c}_{1}\cdot ds{k}_{s}\right) mod\left(q\right)$$

which is implemented into COLLAGENE using the existing decryption function and the modular polynomial matrix subtraction in SEAL as:

$$pardec\left(\left({c}_{0},{c}_{1}\right);ds{k}_{s}\right)=\left(dec\left(\left({c}_{0},{c}_{1}\right);ds{k}_{s}\right)-{c}_{0}\right) mod\left(q\right)$$

After each site calculates partial decryptions, it encrypts the partially decrypted ciphertexts using the common symmetric key (from *KeyMaker*) and shares it among each other (through shared space such as an AWS bucket). This encryption aims at ensuring that outside parties cannot directly access the partial decryptions. This symmetric encryption is implemented using openssl and is independent of CKKS and SEAL.

#### Aggregation of partial decryptions

Each site downloads the partial decryptions of other sites (using network interface) and decrypts the symmetric encryption. Next, the partial decryptions are pooled to obtain the decryption under master secret key:

$$dec\left(ct=\left({c}_{0},{c}_{1}\right);msk\right)=\left({c}_{0}+\sum\limits_{s}pardec\left(\left({c}_{0},{c}_{1}\right);ds{k}_{s}\right)\right) mod \left(q\right)$$

In the aggregation, the summations of the list of partial decryptions are performed using SEAL’s polynomial arithmetic functions. In the aggregation process, each site must also add ${c}_{0}$ term only once for correct decryption. This is implemented into COLLAGENE by allowing only one site to include ${c}_{0}$ in the partial decryptions. This can be alternatively implemented by accessing to the decrypted ciphertext since all sites have access to ${c}_{0}$. After the aggregation, the data is decoded using SEAL::CKKSEncoder class.

Another important aspect of collective decryption is the protection of the partially decrypted data. In the above formulation, the partial decryptions are simple polynomial multiplications, $\left({c}_{1}\cdot ds{k}_{s}\right) mod \left(q\right)$. Any entity who has access to partial decryptions and the ciphertext $\left({{c}_{0},c}_{1}\right)$ can try to divide the partial decryption by ${c}_{1}$ and recover the secret key share of site $s$. To provide protection against these attacks, COLLAGENE uses a large noise term (also referred to as “smudging” or “ciphertext flooding” noise [113]), which is generated from the discrete Gaussian distribution implemented in SEAL library. Given a smudging noise variance in ${n}_{sm}$ bits, i.e., ${\sigma }_{sm}^{2}={2}^{{n}_{sm}}$, a polynomial, denoted by ${e}_{sm}$, is sampled from the clipped normal distribution with variance ${\sigma }_{sm}^{2}$ and added to the partial decryption:

$$pardec\left(\left({c}_{0},{c}_{1}\right);ds{k}_{s}\right)=\left(\left({c}_{1}\cdot ds{k}_{s}\right)+ {e}_{sm}\right) mod\;q$$

For an adversarial entity with access to ${c}_{1}$ and the partial decryption, the goal will be to recover $ds{k}_{s}$ when ${e}_{sm}$ is not known by the adversary. In the above equation, the exponentially large smudging noise statistically obliterates the information leakage from the partial decryption result, i.e., $\left({c}_{1}\cdot ds{k}_{s}\right)$. COLLAGENE utilizes 40 bits of noise by default to increase noise level in the partially decrypted data and the noise variance can be can be modified by the users.

### Matrix representation and arithmetic

#### Matrix encryption

For storing a matrix with $a$ rows and $b$ columns, COLLAGENE first concatenates the rows of the matrix into an array of length $a\times b$ (Fig. 2a). Next, the array is encrypted into $\left\lceil a\cdot\frac b{slot\_size}\right\rceil$ many ciphertexts using the common public key, where $slot\_size$ is equal to half of the polynomial modulus degree. The matrix dimensions and the encrypted ciphertext are written to a binary file. Plaintext matrices are stored with matrix dimensions and the flattened vector.

#### Matrix arithmetic

COLLAGENE has options to perform basic matrix arithmetic operations including secure addition, subtraction, and matrix/scalar multiplication. Elementwise summation, subtraction, and multiplication of matrices are implemented by addition, subtraction, and multiplication of the ciphertexts for the matrices. For matrix multiplications, we adopt a simplification of the approach devised in Jiang et al. [114]: Given two plaintext matrices of sizes $\left(a\times b\right)$ and $\left(b\times c\right)$, we first generate the *column expansion* of the $M1$ and *row expansion* of $M2$, which are basically repetitions of columns and rows of the matrices. Given $M$, an $\left(a\times b\right)$ matrix, the $c$-sized column expansion is an ordered set of $\left(a\times c\right)$ sized matrices ${ce}_{\cdot }^{\left(c\right)}\left(M\right)=\left[c{e}_{i}^{\left(c\right)}\left(M\right), i\le b\right]$, denoted by ${ce}_{i}^{\left(c\right)}\left(M\right)$, is formed by concatenation of the *i*th column of matrix $M$ for $c$ times, i.e., ${col}_{i}^{\left(c\right)}\left(M\right)={\left[{M}_{\cdot ,i} {M}_{\cdot ,i} {M}_{\cdot ,i}\dots {M}_{\cdot ,i}\right]}_{a\times c}$ for $1\le i\le b$. Similarly, the $a$-sized row expansion of a $\left(b\times c\right)$ matrix $M$, ${re}_{\cdot }^{\left(a\right)}\left(M\right)$, is an ordered set of $\left(a\times c\right)$ sized matrices, which are formed by concatenating each of the b rows of $M$, i.e., ${re}_{i}^{\left(a\right)}\left(M\right)={{\left[{M}_{i,\cdot }^{\prime} {M}_{i,\cdot }^{\prime} {M}_{i,\cdot }^{\prime}\dots {M}_{i,\cdot }^{\prime}\right]}^{\prime}}_{a\times c}$ for $1\le i\le b$.

Row and column expansions of plaintext matrices are performed efficiently by copying memory using parallelized calculations. Each matrix in the expansion is encrypted before being saved. Given the column and row expansions of two matrices $M1$ and $M2$, respectively, matrix multiplication of $M1\times M2$ can be written as elementwise matrix products [114]:

$${M1}_{a\times b}\cdot {M2}_{b\times c}=\sum\limits_{1\le i\le b}\left({ce}_{i}^{\left(c\right)}\left(M1\right)\odot {re}_{i}^{\left(a\right)}\left(M2\right)\right)$$

where $\odot$ denotes elementwise matrix multiplication, i.e., ${\left(M1\odot M2\right)}_{i,j}={M1}_{i,j}\cdot {M2}_{i,j}$. Elementwise multiplication is implemented by multiplication of corresponding ciphertexts in $M1$ and $M2$. A useful property of the expansions is that they are distributive over addition (and subtraction), e.g.,

$${re}_{i}^{\left(c\right)}\left(M1+M2\right)={re}_{i}^{\left(c\right)}\left(M1\right)+{re}_{i}^{\left(c\right)}\left(M2\right), \forall i\in \{\mathrm{1,2}, \dots ,b\}.$$

#### Secure row-row multiplication (Fig. 2c)

Inner products are often used in statistical inference. While these can be implemented using row/column expansions, this may impose a performance penalty. To counter this, COLLAGENE implements row-to-row inner products, denoted by ${\langle M1,M2\rangle }_{r2r}$ of two encrypted matrices. Given two encrypted matrices $M1,M2$ with dimensions $a\times b$, row-row multiplication at row $i$ requires calculating the inner product of $M{1}_{i}$ and $M{2}_{i}$, $\langle M{1}_{i},M{2}_{i}\rangle$, and storing this encrypted value at the *i*th entry of the resulting vector. We implement the inner products using a recursive shift-and-add approach that uses ${\mathrm{log}}_{2}\left(b\right)$ rotations by iteratively adding the row-row multiplications after applying circular shifts on the accumulated values:

1. Set the current inner product vector for row $i$ as ${r2r}_{i}=\left({M1}_{i}\odot {M2}_{i}\right)$, and set current rotation to $t=1$.
  The inner product vector is calculated by elementwise multiplication of the ciphertexts in $M1$ and $M2$ that contain row $i$.
2. Update $${r2r}_{i}\leftarrow \left({r2r}_{i}+\left({r2r}_{i}\ll t\right)\right)$$
  In this step, we use the Galois keys to perform rotations by $t$, which circularly rotates the underlying array in the ciphertext by $t$ entries to left. Summations are performed elementwise.
3. Update $$t\leftarrow 2t$$
4. If $t>b$, return ${r2r}_{i}$, otherwise go to step 2.

This algorithm runs for ${\mathrm{log}}_{2}\left(b\right)$ iterations and is efficient even for large $b$. After the inner product vector is calculated, the first entry in it contains the inner product of *i*th rows of $M1$ and $M2$. We copy the first entry in ${r2r}_{i}$ to the *i*th element of the final vector. A useful observation is that when the above iterations are executed on a ciphertext that contains multiple rows, row-row inner products for all of the rows in the ciphertexts are simultaneously calculated at every *b*th entry. After processing one ciphertext with shift-and-add method, we copy every *b*th element in the resulting ciphertext to the final row-row inner product vector. For matrices that are stored in multiple ciphertexts, we repeat this process over all ciphertexts. It is, however, necessary to ensure that the column number $b$ is an exact power of 2, i.e., $b={2}^{n}$ for $\in {Z}^{+}$. This can be satisfied by padding the rows of the matrix with zeros before encryption. Row-row inner products are useful to perform consecutive multiplications of encrypted matrices that involve quadratic forms, i.e., $XWX^{\prime}$, which are commonly used in statistical inference (Fig. 2b).

COLLAGENE uses a similar shift-and-add approach to perform row expansions of encrypted matrices. For this case, each row is copied to a new ciphertext, which is recursively shifted and added to expand the rows to requested size. Finally, the ciphertexts corresponding to the expansion of the current row are saved. This operation is repeated for all the rows in the matrix.

#### Matrix padding, scaling, and random matrix generation

Matrix padding has numerous important applications for secure matrix arithmetic. COLLAGENE has functions to pad plaintext matrices with zero values in rows and columns to custom and specific sizes (e.g., row/column numbers are powers of 2) makes it convenient to process matrices to specific shapes, i.e.,

$$pad\left(M,c,d\right)={M}_{c\times d}^{\prime}={\left[\begin{array}{cc}{M}_{a\times b}& {0}_{a\times \left(d-b\right)}\\ {0}_{\left(c-a\right)\times b}& {0}_{\left(c-a\right)\times \left(d-b\right)}\end{array}\right]}_{c\times d}$$

For processing matrices with large dynamic ranges, scaling the matrices increases numerical accuracy. For example, this is observed while padding a matrix that contains entries close to zero if the matrix will be multiplied with another matrix with larger values. To counter these, COLLAGENE has number of functions to make it convenient to scale matrices. Finally, COLLAGENE implements options to generate random matrices that can be used as noise to hide data while performing certain intermediate steps, e.g., matrix inversion, efficiently (Fig. 2d).

### Network I/O for exchanging intermediate encrypted matrices

After each site performs secure calculations and wants to share encrypted intermediate results, the data is written into an encrypted matrix file and sent over the network to the shared storage space. COLLAGENE currently implements a separate network module that can be configured to use 3 options: First is the “local” option, where the files are stored in a local disk. This option is used for simulating collaborative analysis without any network traffic and can be used for development or debugging protocols. Second option is the “SCP” option where sites can configure an FTP/SFTP server to store the files for executing the protocols. This option utilizes *scp* command line tool for uploading/downloading and probing files. Third option is “S3” that utilizes an AWS S3 bucket to store the encrypted intermediate matrix files. This option uses the AWS command line interface utilities for file transfers (upload, download, probe, and wait).

### Federated GWAS for binary phenotypes

We describe the specific matric operations federated GWAS calculations that utilize COLLAGENE’s functionalities. We denote the number of sites with $S$, the number of variants with $M$, and sample size with ${N}_{s}$ for $s\le S$, and the number of covariates with $p$, which includes an intercept by default.

The null model fitting starts with the covariate matrix ${X}_{s}$, binary phenotype vector ${y}_{s}$, and the initial null model weights, $\alpha =0$. We assume that the sites setup an SCP file server or use an AWS S3 bucket to be used for storing the encrypted intermediate files. Following is calculated at each site:

1. Calculate $${\mu }_{0,s}=sigmoid\left({X}_{s}\cdot \alpha \right)$$, where $$sigmoid\left(a\right)=\frac{{e}^{-a}}{1+{e}^{-a}}$$
2. Calculate $${W}_{s}=diag\left({\mu }_{0,s}\cdot \left(1-{\mu }_{0,s}\right)\right)$$
3. Calculate $${z}_{s}={X}_{s}\cdot \alpha +\frac{\left({y}_{s}-{\mu }_{0,s}\right)}{{\mu }_{0,s}\cdot \left(1-{\mu }_{0,s}\right)}$$
4. Calculate ${\Upsilon }_{s}={X}_{s}^{\prime}{W}_{s}{X}_{s}$ ($p\times p)$ and ${X}_{s}^{\prime}{W}_{s}{z}_{s}$ ($p\times 1$) at each site $s\le S$.
5. Each site generates a ($p\times p$) masking matrix ${H}_{s}$ by sampling unit Gaussian random variable, calculates the encrypted size-$p$ row expansion ${re}_{i}^{\left(p\right)}\left({H}_{s}\right)$, and uploads the expansions to the shared space. Each site also pads the masking matrix to set the size to be equal to the closest power-of-2, i.e., $pad\left({H}_{s}, {2}^{\lceil{\mathrm{log}}_{2}(p)\rceil}, {2}^{\lceil{\mathrm{log}}_{2}(p)\rceil}\right)$. Each site then calculates the size-${2}^{\lceil{\mathrm{log}}_{2}(p)\rceil}$ column expansion of the padded noise matrix, i.e., ${ce}_{i}^{\left({2}^{\lceil{\mathrm{log}}_{2}(p)\rceil}\right)}\left(pad\left({H}_{s}\right)\right)$. The encrypted column expansions of the padded noise matrix are uploaded to the shared server.
6. Sites download the encrypted row and column expansions from the shared server and calculate the total expansions of the masking matrix:
  - ${re}_{i}^{\left(p\right)}\left(H\right)={\sum }_{s\le S}{re}_{i}^{\left(p\right)}\left({H}_{s}\right)$, where $H$ is the total encrypted noise matrix that includes noise from all sites.
  - $${ce}_{i}^{\left({2}^{\lceil{\mathrm{log}}_{2}(p)\rceil}\right)}\left(pad\left(H\right)\right)={\sum }_{s\le S}{ce}_{i}^{\left({2}^{\lceil{\mathrm{log}}_{2}(p)\rceil}\right)}\left(pad\left({H}_{s}\right)\right)$$
  - Calculate the column expansion of ${\Upsilon }_{s}$, i.e., ${ce}_{i}^{\left(p\right)}\left({\Upsilon }_{s}\right)$ and calculate the multiplication of ${\Upsilon }_{s}$ with $H$: ${\Upsilon }_{s}H={\sum }_{i\le p}\left({ce}_{i}^{\left(p\right)}\left({\Upsilon }_{s}\right)\odot {re}_{i}^{\left(p\right)}\left(H\right)\right)$, and uploads encrypted ${\Upsilon }_{s}H$ to shared server.
7. Sites download ${\Upsilon }_{s}H$ for all $s\le S$ from the shared server and securely add them to calculate the total masked and encrypted $\Upsilon H={\sum }_{s}\left({\mathrm{\Upsilon}}_{s}H\right)$.
  - This matrix is collaboratively decrypted: $coldec(\Upsilon\mathrm H)$, which reveals ${\Upsilon} H$, $\left({X}^{\prime}WX\right)H$ to all sites. At this point, each site inverts the ${\Upsilon}\mathrm{H}$ in plaintext form to calculate ${H}^{-1}{\mathrm{\Upsilon}}^{-1}={H}^{-1}\left({X}^{\prime}WX\right)$. Each site pads this inverted masked matrix and calculates the row expansion of the padded inverted matrix, ${re}_{i}^{\left({2}^{\lceil{\mathrm{log}}_{2}\left(p\right)\rceil}\right)}\left(pad\left({H}^{-1}{\Upsilon }^{-1}\right)\right)$
  - Finally, each site removes the masking noise ${H}^{-1}$ securely using the row in Step 7a and column expansion in Step 6b:
    $$pad\left({\Upsilon }^{-1}\right)=\sum\limits_{i\le {2}^{\lceil{\mathrm{log}}_{2}(p)\rceil}}\left({ce}_{i}^{\left({2}^{\lceil{\mathrm{log}}_{2}(p)\rceil}\right)}\left(pad\left(H\right)\right)\odot {re}_{i}^{\left({2}^{\lceil{\mathrm{log}}_{2}\left(p\right)\rceil}\right)}\left(pad\left({H}^{-1}{\Upsilon }^{-1}\right)\right)\right)$$
8. We now update $\alpha$. First each site downloads encrypted and padded $pad\left({X}_{s}^{\prime}{W}_{s}{z}_{s},{2}^{\lceil{\mathrm{log}}_{2}\left(p\right)\rceil},1\right)$ from the shared server and securely sums these vectors among sites: $X^{\prime}Wz={\sum }_{s}\left(pad\left({X}_{s}^{\prime}{W}_{s}{z}_{s},{2}^{\lceil{\mathrm{log}}_{2}\left(p\right)\rceil},1\right)\right)$, which is a $\left({2}^{\lceil{\mathrm{log}}_{2}\left(p\right)\rceil}\times 1\right)$ vector. Each site generates the encrypted row expansion of padded $\left({X}^{\prime}Wz\right)^{\prime}$, i.e., $r{e}_{i}^{\left({2}^{\lceil{\mathrm{log}}_{2}\left(p\right)\rceil}\right)}\left(pad\left({X}^{\prime}Wz\right)^{\prime}\right)$, which has only one matrix in it since it is a row vector. We finally use the row-row multiplication to calculate the updated the null model parameter estimate in encrypted form:
  $${\alpha }_{new}={\langle pad\left({\Upsilon }^{-1}\right), r{e}_{0}^{\left({2}^{\lceil{\mathrm{log}}_{2}\left(p\right)\rceil}\right)}\left(pad\left({X}^{\prime}Wz\right)^{\prime}\right)\rangle }_{r2r}$$
9. Sites collectively decrypt ${\alpha }_{new}$ and use it in a new update.
10. Move back to step 1.

The main ingredient of the null model fitting is inversion of $\left({X}^{\prime}WX\right)$ in plaintext domain after hiding it with collective mask. This step aggregates a mask matrix from all sites and the pooled mask matrix is multiplied with $\left({X}^{\prime}WX\right)$. This multiplication is performed in secure domain and hides covariate covariance values. We thus make use of the masking to simplify matrix inversion. It should be noted that the inversion can be performed fully in secure domain using an implementation of the Gauss-Jordan inverse, which has ${n}^{3}$ time complexity. But this method has a large multiplicative depth and becomes infeasible with 10–15 covariates and requires large number of ciphertext refreshes or bootstraps.

The null model fitting is an implementation of the IRLS algorithm [115] that calculates ${\alpha }_{new}={\left({X}^{\prime}WX\right)}^{-1}\cdot ({X}^{\prime}Wz)$ using the current estimate ${\alpha }_{current}$ to calculate $W$ and $z$ in the current iteration. An important aspect is that covariate data $X$ is always in aggregated form (via matrix products with $W$) and is stored in small dimensions ($p\times p$) as masked and/or encrypted form. We reformulated the inference steps for federated scenario by partitioning the matrix multiplications and implemented the algorithm using COLLAGENE’s modules. In the above formulations, all aggregations (which we refer to as pooling in figures) are implemented by secure elementwise matrix additions. Matrix multiplications, including row and column expansions and row-row inner products, are described in matrix operations.

#### Assignment of p-values

We use the GMMAT’s score test to assign the *p*-values using the null model predicted phenotypes to all subjects:

1. Calculate $${T}_{s}={G}_{s}^{\prime}({y}_{s}-{\mu }_{0,s})$$  
2. Calculate $${G}_{s}^{\prime}{W}_{s}{G}_{s}$$  
3. Calculate ${G}_{s}^{\prime}{W}_{s}{X}_{s}$, do column expansion on it and multiply it with $pad({\Upsilon }^{-1}, {2}^{\lceil{\mathrm{log}}_{2}\left(p\right)\rceil},{2}^{\lceil{\mathrm{log}}_{2}\left(p\right)\rceil})$ (computed in step 7b) on the right using its row expansion. This multiplication yields the padded version of $\left({G}_{s}^{\prime}{W}_{s}{X}_{s}{\Upsilon }^{-1}\right)$. These matrices are uploaded to the shared working space.
4. Each site downloads ${T}_{s}, {G}_{s}^{\prime}{W}_{s}{G}_{s}, {G}_{s}^{\prime}{W}_{s}{X}_{s}$, and $\left({G}_{s}^{\prime}{W}_{s}{X}_{s}{\Upsilon }^{-1}\right)$ for all $s\le S$, and securely pools them across all sites: $T={\sum }_{s}{T}_{s}$, ${S}_{1}={\sum }_{s}{G}_{s}^{\prime}{W}_{s}{G}_{s}$, ${S}_{21}={\sum }_{s}{G}_{s}^{\prime}{W}_{s}{X}_{s}$, ${S}_{22}={\sum }_{s}\left({G}_{s}^{\prime}{W}_{s}{X}_{s}{\Upsilon }^{-1}\right)$.
  - It should be noted that these are encrypted matrices and ${S}_{21}$ and ${S}_{22}$ are padded to the next power of 2. Each site performs row-row multiplication of these: ${{S}_{2}=\langle {S}_{21},{S}_{22}\rangle }_{r2r}$
  - $S={S}_{1}-{S}_{2}$ is calculated as the scale parameter of the chi-squared distribution for the *p*-values.
5. We cannot decrypt $T$ and $S$ to assign the *p*-values because $T$ can leak genotype information and cannot be decrypted without privacy concerns. To get around this issue, we propose using a multiplicative mask that conserves $\frac{T}{S}$ ratio but hides the actual values of $T$ and $S$ statistics before collective decryption.
  - Each site samples a random noise value and multiplies $T$ and $S$ statistics using the same sampled noise value and uploads the noisy $T$ and $S$ statistics to the shared space.
6. All sites download the noisy $T$ and $S$ statistics from the shared space and pool them among all sites. The final noisy $T$ and $S$ statistics that contain the noise levels from all sites are collectively decrypted. The *p*-values are assigned using the asymptotic null distribution of $\frac{T}{S}$ statistic, which is a chi-squared distribution with 1-degree-of-freedom.

It should be noted that this protocol requires sites to adhere to the protocol without malicious deviations from the protocol. Any malicious deviation can result in either corrupted data or decryption of data that is not intended to be decrypted.

### Secure meta-analysis of GWAS results among multiple sites

Given GMMAT results with $S$ and $T$ statistics, we implement the secure meta-analysis by first pooling of masked $\frac{T}{S}$ statistics from each site. The masked statistics are pooled and collectively decrypted. As GMMAT can integrate random effects into analysis, meta-analysis provides a very efficient way of combining summary statistics in a secure manner while accounting for complex random effects such as kinship [116].

### Estimation of per-thread throughput for sPLINK

Nasirigideh et al. [74] reported sPLINK finished a 3-site federated GWAS for 580,000 SNPs in 75 min. Each client used 4 threads with total of 12 threads. sPLINK also utilizes 2 more sites, i.e., Compensator and Aggregator sites that used 8 and 4 threads, respectively. In total, all sites (including clients, Aggregator, and Compensator) utilized 24 threads. We therefore estimate the per-thread throughput of sPLINK as:

$$\left(\frac{\mathrm{580,000}\frac{SNPs}{site}\times 3\, sites}{75\, minutes}\times 60\, minutes\right)\times \frac{1\, thread}{24 \,thread}\approx \mathrm{58,000}\, SNPs$$

We acknowledge that the above formula may underestimate the actual throughput since all threads are not utilized with 100% efficiency. However, the CPUs are allocated for the algorithm, and we believe this is a fair estimate that reflects the overall algorithmic efficiency for sPLINK.

### Data simulations

#### Simulated genotype-phenotype datasets

We obtained the VCF formatted genotype datasets and the population information from the 1000 Genomes Project Portal (see “Availability of data and materials”).

##### Genotype data simulation

We separated the 1000 Genomes panel with respect to three super-population-based cohorts; specifically, we used EUR (European), AFR (African), and EAS (East Asian) subjects. Next, we uniformly subsampled the variants down to 57,344 variants to decrease computational requirements. Next, each cohort was separated into a distinct genotype file. Each super-population cohort is used to simulate the genotype dataset for a corresponding site, i.e., for the 3 sites that are collaborating. For each super-population cohort, we sampled genotypes of the 57,344 variants for 4800 individuals. This sampling is done without accordance to the linkage-disequilibrium (LD) structure. We used a pedigree-based sampling, which was not explicitly used in the analysis.

##### Phenotype data simulation

Among 4800 subjects at each site, we first assigned gender such that half of the cohort was assigned female and remaining half is set to male subjects. Next, we extracted the covariates by projecting the samples on the principal components (PCs) that are calculated from the original pooled EUR, AFR, and EAS sample set. We used the top 8 PCs and these projected coordinates were used as fixed covariates for population-level stratification in GWAS calculations. Next, we generated a random model that used 20 random SNPs as causal variants for which the effect size was set by randomly sampling uniform random variable, i.e., $\beta \sim U(-0.5, 0.5)$. The gender was assigned a constant effect size of 0.1. This model was used first used to calculate the linear combination of all covariates and genotype effect sizes for all individuals in the 3 sites using following relationship:

$${\eta }_{i}=\left(\sum\limits_{j}{G}_{ij}{\beta }_{j}\right)+{X}_{i}{\alpha }_{gender}+{\epsilon }_{i}$$

$\epsilon$ denotes the environmental noise component and is sampled from $\epsilon \sim N(\mathrm{0,0.5})$. Here, ${X}_{i}$ includes only the gender for simulating the phenotype. We finally mapped the linear combinations using a logistic function to assign the final simulated phenotypes:

$${Y}_{i}=\left\{\begin{array}{c}1,\;if\, logistic\left({\eta }_{i}\right)>0.5\\ 0,\;if\, logistic\left({\eta }_{i}\right)<0.5\end{array}\right.$$

#### LOAD dataset

LOAD data was acquired from dbGAP under accession identifier phs000168.v2.p2 that contains 571,166 variant genotypes for 3007 subjects, among which 2545 subjects had an assigned AD diagnosis phenotype. We used the first 557,056 variants in the dataset which can be loaded exactly into 68 blocks of ciphertexts (i.e., 68 blocks of 8192 variants). The subject identifiers were shuffled and split among 3 sites for simulating collaborative analysis. Plink2 analysis was performed using “—glm” option to calculate *p*-values for each variant using the pooled dataset. GMMAT was run with default parameters. It should be noted that the LOAD dataset is under restricted access and we are not allowed to share it publicly.

### Supplementary Information

**Additional file 1. **Review history.

## Data Availability

Simulated genotype-phenotype datasets We downloaded the VCF formatted genotype datasets from the 1000 Genomes Project [117] Portal [118] at https://ftp-trace.ncbi.nih.gov/1000genomes/ftp/phase1/analysis_results/integrated_call_sets/. The population-level information is obtained from the 1000 Genomes Project Portal from ftp://ftp.1000genomes.ebi.ac.uk/vol1/ftp/technical/working/20130606_sample_info/20130606_sample_info.xlsx. LOAD data can be accessed from database of Genotypes and Phenotypes (dbGAP) via accession identifier phs000168.v2.p2. This dataset is distributed under a restricted access data usage agreement and requires approval by dbGAP before accession. The simulated datasets supporting the conclusions of this article are available in the Zenodo repository (DOI 10.5281/zenodo.8106630 [119]) at following link: https://zenodo.org/record/8106630/files/CLLGN_DATA_DIR_07_02_23_20_00_40.tar.bz2?download=1 These data can be downloaded and extracted using the following commands: *wget -c* https://zenodo.org/record/8106630/files/CLLGN_DATA_DIR_07_02_23_20_00_40.tar.bz2?download=1 This directory contains the simulated federated GWAS and meta-analysis datasets that can be readily run after installing COLLAGENE. The latest version of COLLAGENE is available under open source MIT license at https://github.com/harmancilab/COLLAGENE [120]. It can be downloaded from the GitHub repository using following command: git clone https://github.com/harmancilab/COLLAGENE.git The code, documentation, and usage examples are included in the repository. The code that is used in the manuscript is separately archived in Zenodo (DOI 10.5281/zenodo.8125935 [121]), which can be accessed through https://zenodo.org/record/8125935.
