# Supplementary material for: COLLAGENE enables privacy-aware federated and collaborative genomic data analysis
Source: Genome Biol. 2023 Sep 11;24:204. doi: 10.1186/s13059-023-03039-z (PMC10496350; doi:10.1186/s13059-023-03039-z)
Supplement: Supplementary file 1 — Additional file 1. Review history. [file 13059_2023_3039_MOESM1_ESM.docx]

**1^st^ round**

**Reviewer 1**

-Is the Software likely to be of broad utility? Is it easy to install and use?
Privacy-preserving analysis frameworks for multi-party access in the context of genomic data are certainly of interest to researchers given increasing regulatory strictness around such data. Thus, the software is potentially of broad utility. However, it should be emphasized more strongly, that the privacy guarantees of the tool are based on an honest-but-curious scenario and do not protect from malicious intent. No comment can be made on the useability of the tool, since the provided repo only contains a readme.
-Does the software represent a significant advance over previously published tools (as demonstrated by direct comparison with available related software)?
While the software mostly recycles functionalities from already existing tools, it advertises better utility by providing a use-friendly wrapper. Given the lack of access to the tool (see point above), we cannot provide feedback on that.
-Please indicate briefly the novel features and/or advantages of the software and/or please reference the relevant publications and which alternatives, if any, it should be compared with.
The software offers a combination of an available programming interface together with custom command line tools. It further focuses on allowing secure matrix operations to make it compatible with the a common data representation in bioinformatics. It does not require a central instance that comes into contact with sensitive data, avoiding a vulnerable target for attacks.
-Are the methods appropriate to the aims of the study, are they well described, and are necessary controls included?
yes
-Are the conclusions adequately supported by the data shown?
yes
-Are sufficient details provided to allow replication and comparison with related analyses that may have been performed?
No, since the Alzheimer's dataset version (v2, only v1.1 can be found) cannot be found and the tool is not accessible. If both were accessible, the answer would be yes.
-Is the paper of broad interest to others in the field, or of outstanding interest to a broad audience of biologists?
The paper could be of broad interest to others in the field.

Minor points:
- page 4, line 48: word "necessary" duplicated.
- page 4, line 54ff: remove pseudo-heading "Matrix Masking by Additive and Multiplicative Noise",since it disrubts the enumaration of "first" and "second".
- page 5, line 4: please rephrase "fresh"
- page 9, line 30ff: network traffic is reported from 1.6gb to 4.5gb (3x change) but in line 32 and expansion rate of 15 is mentioned, please clarify.
- page 9, 44ff: "This indicates that the network traffic takes almost half of the computation time" - this is not clear from the presented data.
- page 12, line 19: word "are" duplicated.
- page 12 line 34: Reference to Homer et al. missing
- Legend figure 4: difference between e-h and i-l is not clear, please describe
- Legend figure 4: texts for i,j and k,l are dirct copies of each other
- Figure 4 b,d: please remove legends ("color red")
- Figure 4: The different plots are hard to compare since they are so spread out. Consider choosing a layout that allows better comparison of the presented results. Either plot corresponding plot next to each other or, better, plot them in the same plot.
- Figure 5 a,b: please add Spearman R2 like in Fig 4 a-d

**Reviewer 2**

The authors have introduced an integrated secured federated learning library that combines homomorphic encryption (HE) and differential privacy (DP) to enhance privacy preservation in federated training using GWAS data. Additionally, their proposal of an efficient GMMAT scoring method adds novelty to the methodology. In all, this study introduces a novel privacy-preserving federated learning scheme called COLLAGENE, which holds promise for various applications in different domains beyond GWAS data analysis. The framework's potential contribution extends to broader areas, making it an intriguing research endeavor.


Positives:
The authors have conducted a thorough investigation of state-of-the-art secured federated learning studies, highlighting their advantages and limitations. The utilization of null-model fitting and GMMAT scoring has demonstrated performance improvements compared to existing logistic regression models in the context of federated learning. Furthermore, the paper provides detailed optimization strategies for both software and hardware aspects, making the COLLAGENE framework efficient. The methodology is well-presented, and the framework is open source, which enhances the reproducibility of this work.

Negatives:
It is commendable that the paper includes a detailed analysis and comparison of the time and resource costs associated with the proposed method. However, the manuscript can be improved by adding clarifications to their methodology and results. For example, the manuscript would benefit from providing explanations or formulas regarding the aggregation approach, and specifications of the strategy employed for the aggregation process. In the section of "Comparison on Real data", more details should be added to summarize the observations based on figures (e.g. Fig. 4.c and 4.d).

**Authors’ response**

Reviewer #1

**Reviewer’s Comment:** *Is the Software likely to be of broad utility? Is it easy to  install and use?* ***Privacy-preserving analysis frameworks for multi-party access  in the context of genomic data are certainly of interest to researchers given  increasing regulatory strictness around such data. Thus, the software is  potentially of broad utility.***

**Authors’ Response:** We thank the reviewer sincerely for reviewing our study. We have addressed all  comments to the best of our ability.

**Reviewer’s Comment:** *1) "****However, it should be emphasized more strongly, that the  privacy guarantees of the tool are based on an honest-but-curious scenario and  do not protect from malicious intent. ..."***

**Authors’ Response:** We thank the reviewer for raising this important distinction that was not clearly conveyed  in our original submission. We acknowledge that COLLAGENE does not provide security against malicious  entities. We believe that the majority of the adversarial entities in the biomedical research community can be  considered as honest-but-curious entities[1] who are not actively trying to break protocols to steal individual

level information.

The sources of re-identification risks stem mainly from two sources: Firstly, the data may itself lend to  *accidental leakages*[2,3] wherein a researcher may accidentally re-identify a study participant, e.g., linking two  datasets may pinpoint an individual’s unique identifying information in a third dataset[4,5]. Although the risk  may seem low, the increasing number of high dimensional datasets may render these cases possible (e.g.,  knowledge of a handful of ultrarare variants may reveal an individual’s participation in a database). In this  scenario, the researcher is not acting maliciously, or even in a curious manner. Accidental leakages can be  easily thwarted by using a privacy-aware study design. This aligns exactly with the COLLAGENE’s design and  framework.

Secondly, the most prevalent privacy concerns in genomics and biomedical literature stem from re identification concerns such as the scenarios laid out by Latanya Sweeney’s linking attack[4,5], Gymrek et  al.’s demonstration of the linking genomics to genealogical datasets[7], Homer et al t-test[8] and  Sankararaman’s likelihood ratio[8] test, and Bustamante’s attack[9] which are relatively easy to apply so long  as the large-scale summary statistics are available from the data, even without access to the raw datasets.  Due to their simplicity, they can be applied by honest-but-curious entities, without actively breaking protocols. In COLLAGENE’s framework, the decrypted intermediate statistics (for ciphertext refreshes, for example), may  be used for performing these attacks.

To counter these attacks, we reiterate our suggestions for protocol design: We advise the users minimize the  intermediate data dimensions and increase aggregation property. Consequently, protocols should not decrypt  large dimensional intermediate results. For example, masking should not be applied to individual level raw  data if the masked matrices will be decrypted as intermediate data processing step; masking should always  be applied on aggregated datasets that are calculated using preferably non-linear aggregation functions

because high dimensional individual-level data matrices can be attacked even when they are masked [10–12] Of note, these attacks require specific conditions to be satisfied and they are technically challenging to  execute. It is worth noting that powerful and highly specific re-identification requires leakage of large amount of re-identifying information (e.g., large number of common features or small number of rare features is  sufficient) and the statistical properties of features should be considered while decrypting intermediate  datasets. These matrices should never be decrypted unless they are masked at the aggregate level. We  believe these suggestions are reasonable since privacy-aware federated learning algorithms almost always  share aggregated datasets and almost never share individual level datasets.

We discuss some of the more complex scenarios that can involve malicious entities:

**Some datasets may be selectively targeted by malicious adversaries.** We also would like to note that  certain genomic datasets, e.g. rare disease genomes, underrepresented populations, genomic data from  individuals with stigmatizing conditions, may be more actively targeted by malicious entities for malicious  purposes. The users should be cautious while processing these datasets if there is any concern around  leakage from decrypted datasets. The algorithms that process these data must strictly adhere to the previous  rules we laid out.

**Large-scale collaborations increased attack surfaces.** The following threat stems from collaborations  among large number of entities, e.g., a crowdsourcing approach. Although COLLAGENE’s current model  relies on the idea that collaborating entities provide mutual trust for scientific collaboration, increasing the  number of collaborating sites broadens the attack surface and may make the partial and full decryptions  available to unauthorized malicious entities. In these cases, the partial decryptions should be protected by  symmetric encryption key that *KeyMaker* supplies to the sites.

**Malicious Parties may deviate from protocols.** Another more complex threat is actively “sabotaging” collaborative analysis. In these attacks, malicious entities try to cripple the analysis by injecting pure noise or  by deviating from agreed protocols. The protection against these adversarial efforts requires entities to verify  computations performed on the data using, for example, protocol verification. This is a very complex task that  requires computationally expensive online protocols. We assume these attacks are outside the focus of  protocols that COLLAGENE supports.

**Final Results are always decrypted and should be protected by data access policies.** It is important to  note that the final results are always decrypted and therefore will always be revealed to the collaborating  parties. For example, the decrypted GWAS summary statistics may be used for Homer’s attacks that rely on  GWAS summary statistics to reveal participation of an individual in the collaborative study. Although this attack  would not immediately re-identify which site contains the target individual, this risk should be accounted for  based on the trust and data access policies among the participants for ensuring that the collaborating entities

do not use the data for any unintended purposes. From this perspective, we believe these are out of scope of  COLLAGENE’s codebase because COLLAGENE provides the means for protecting the data in the course of  collaborative analysis workflows.

**Protection against malicious adversaries is very challenging.** Due to the sheer size of aforementioned malicious attack scenarios, theoretically provable protection against malicious entities is very challenging and  occasionally intractable. Notably, privacy protection against malicious entities is an open field of research that  combines data access validation, protocol verification, extensive access control requirements[13]. These  approaches may massively increase computational requirements and protocol complexity which in turn may  degrade the incentives for collaborative research.

We have updated the main text with a summarization of the above points and with an extended discussion to  describe the adversarial entities that COLLAGENE can protect against.

**Reviewer’s Comment:** *"...No comment can be made on the useability of the tool,  since the provided repo only contains a readme.*

**Authors’ Response:** We have now updated COLLAGENE’s public GitHub repository at (www.github.com/harmancilab/COLLAGENE) with documentation, source code, examples, and the use  cases.

We added a markdown to every directory to include extensive documentation for all aspects of COLLAGENE,  including step-by-step installation instructions, CLI usage documentation, publicly available docker  environment, and usage examples.

The usage examples contain 11 introductory examples that users can utilize to build new pipelines. These  examples start from the basic concepts of key generation among sites by interfacing *KeyMaker*, basic secure  matrix arithmetic, collaborative decryption using key shares, collaborative function approximation, and usage  of network I/O modules, among others. These examples are implemented in bash scripts and require no  programming (other than bash scripting) after COLLAGENE is compiled.

The use cases contain the bash script implementations of GWAS and meta-analysis using basic primitives and services that COLLAGENE provides.

**Reviewer’s Comment:** *-Does the software represent a significant advance over  previously published tools (as demonstrated by direct comparison with available  related software)? "****While the software mostly recycles functionalities from  already existing tools, it advertises better utility by providing a use-friendly wrapper. Given the lack of access to the tool (see point above), we cannot  provide feedback on that.****”*

**Authors’ Response:** We thank the reviewer for giving us an opportunity to update the source code and  describe the differences from existing libraries. As we described in our previous response, the source code,  and documentation are now publicly available at www.github.com/harmancilab/COLLAGENE.

Our main motivation for building COLLAGENE is to provide an alternative to most of the current existing  libraries that can be used to implement collaborative and federated analysis pipelines. A justification for this is  the obvious lack of adoption of HE and other privacy mechanisms in the biomedical community. For example,  SEAL is one of the oldest and most well-established libraries but its application domain in bioinformatics is  limited to performing simple tasks, it also does not support multi-party key encryption/decryption as of version  4.1. We believe that one of the main factors for non-adoption in biomedical community is the lack of access to  the documentation and code that can be immediately deployed for building pipelines. For example, in  bioinformatics community, most applications are adopted based on their ease of integration into existing  pipelines with easy learning curve. COLLAGENE aims at encapsulating basic matrix operations so that non experts can immediately start using these even for simple tasks such as aggregating intermediate statistics  among sites (variant counts, calculating means, variances, applying statistical tests) or building more complex  pipelines (OLS, logistic regression, statistical tests).

We divide the current approaches into 2 groups: First are the low-level implementations (e.g., SEAL,  TenSEAL, OpenFHE, Lattigo) that provide complete control on the parameter settings and algorithm design. These methods are developed in various programming languages (SEAL and OpenFHE in C++, Lattigo in Go,  TenSEAL in python, and recently developed SEQURE in a python-like language) that add onto the barriers of  adoption. There is also a lack of standardization even among libraries that implement the same schemes in  terms of parameter names, nomenclatures, and implementations. It becomes extremely burdensome to modify  the low-level libraries without strong familiarity of the software architecture of the libraries. Furthermore, these  libraries are not immediately accessible to non-expert scientists due to lack of technical documentations, which  is understandable, since some of these are still under development. SEAL, which is arguably one of the most  adopted and most well-developed libraries, does not contain complete API documentation. The users need to  learn to program in SEAL using the comments in C++ source code of the exercises with the required expertise  of the RLWE-based cryptographic schemes.

Second group of frameworks focus on federated training of machine learning models, mostly with a focus on neural networks (e.g., PySyft, FedML, OpenFL, Flower). These frameworks aim at capitalizing the community’s need for building ML models using large datasets through collaborations. Methods in this class tend to integrate privacy constraints as a separate layer (in most rigorous cases HE and differential privacy mechanisms) for protecting the model parameter gradients while they are aggregated at a central server and make use of single key approaches when HE is used. It is challenging to decipher how and what type of privacy protection is provided by the frameworks because it is embedded into the model building steps. Some libraries use only end-to-end encryption wherein the shared parameters are decrypted at the clients. These libraries may leak information in the intermediate statistics to an honest-but-curious entity. We place COLLAGENE in between these two groups of approaches that can be a useful alternative: COLLAGENE provides multi-key capability, does not require using complex programming APIs, and bash scripts can be used for seamless integration into existing pipelines. We do acknowledge that not all pipelines are written in bash scripts. This can, however, be circumvented by using system calls in, for example, R or other languages. More expert users are free to modify and use the low level C/C++ implementations of the key sharing and matrix functions and optimize them for their needs. Finally, we also acknowledge that more future work is still necessary to make these tools more available and more adoptable in the biomedical science community.

Reviewer’s Comment: --*Please indicate briefly the novel features and/or advantages of the software and/or please reference the relevant publications and which alternatives, if any, it should be compared with. "The software offers a combination of an available programming interface together with custom command line tools. It further focuses on allowing secure matrix operations to make it compatible with the a common data representation in bioinformatics. It does not require a central instance that comes into contact with sensitive data, avoiding a vulnerable target for attacks."*

Authors’ Response: We agree with the reviewer, COLLAGENE extends the SEAL library to enable a collaborative decryption protocol (which is not implemented in current SEAL distribution) using KeyMaker. As the reviewer points out, KeyMaker does not come into contact with sensitive data. We further combine collaborative decryption library with a matrix library and network file I/O utilities (i.e., uploads, downloads, probing files, waiting files to be ready in the shared space) into ready-to-use command line tool implementations. Overall, the combined set of utilities and strategies (e.g. matrix masking) presented in the manuscript help develop new pipelines with as small a programming footprint as possible. With the included documentation and examples, we hope that COLLAGENE can help lower the level of necessary expertise in using HE in collaborative studies.

Reviewer’s Comment: --Are the methods appropriate to the aims of the study, are they well described, and are necessary controls included?

R1.5. yes

-Are the conclusions adequately supported by the data shown?

R1.6. yes

-Are sufficient details provided to allow replication and comparison with related analyses that may have been performed? R1.7.

"No, since the Alzheimer's dataset version (v2, only v1.1 can be found) cannot be found and the tool is not accessible. If both were accessible, the answer would be yes."

-Is the paper of broad interest to others in the field, or of outstanding interest to a broad audience of biologists?

R1.8. The paper could be of broad interest to others in the field.

Authors’ Response: We have now made the simulated datasets publicly available that can be downloaded from Zenodo scientific data repository with DOI 10.5281/zenodo.8106630. The full URL for the dataset is:

https://zenodo.org/record/8106630/files/CLLGN_DATA_DIR_07_02_23_20_00_40.tar.bz2?download=1

Regarding the dbGAP dataset, we used the study accession “phs000168.v2.p2”. The manifest data for this study defines the genotype and phenotype data as:

“phg000049.v2.LOAD_610K_CIDR_NCRAD.genotype-calls-matrixfmt.c1.GRU.tar”,

“phs000168.v2.pht000707.v2.p2.c1.LOAD610K_Subject_Phenotypes.GRU.txt.gz”, respectively.

These datasets were used in our analysis.

Unfortunately, we are not able to make the Alzheimer’s dataset available since it is under restricted access from dbGAP database

Reviewer’s Comment:

R1.9.- page 4, line 48: word "necessary" duplicated.

R1.10.- page 4, line 54ff: remove pseudo-heading "Matrix Masking by Additive and Multiplicative Noise",since it disrubts the enumaration of "first" and "second".

R1.11.- page 5, line 4: please rephrase "fresh"

R1.12.- page 9, line 30ff: network traffic is reported from 1.6gb to 4.5gb (3x change) but in line 32 and expansion rate of 15 is mentioned, please clarify.

R1.13.- page 9, 44ff: "This indicates that the network traffic takes almost half of the computation time" - this is not clear from the presented data.

R1.14.- page 12, line 19: word "are" duplicated.

R1.15.- page 12 line 34: Reference to Homer et al. missing

R1.16.- Legend figure 4: difference between e-h and i-l is not clear, please describe

R1.17.- Legend figure 4: texts for i,j and k,l are dirct copies of each other

R1.18.- Figure 4 b,d: please remove legends ("color red")

R1.19.- Figure 4: The different plots are hard to compare since they are so spread out. Consider choosing a layout that allows better comparison of the presented results. Either plot corresponding plot next to each other or, better, plot them in the same plot.

R1.20.- Figure 5 a,b: please add Spearman R2 like in Fig 4 a-d

Authors’ Response: We thank the reviewer for careful reading and for their patience, which we greatly appreciate.

R1.9. Removed extra word “necessary” on page 4.

R1.10. Removed the pseudo-heading "Matrix Masking by Additive and Multiplicative Noise" on revised page 5.

R1.11. We rephrased the broken sentence as: “…the matrix is encrypted into a “fresh” ciphertext…” on revised page 5.

R1.12. This point indeed requires further clarification as our original explanation was not exact (revised page 9). The expansion rate of encryption refers to the increase in storage required for ciphertexts compared to the underlying plaintext data, i.e., given a matrix and its encryption,

𝑒𝑥𝑝𝑎𝑛𝑠𝑖𝑜𝑛 𝑟𝑎𝑡𝑒 = 𝑠𝑖𝑧𝑒 𝑜𝑓 𝑒𝑛𝑐𝑟𝑦𝑝𝑡𝑒𝑑 𝑚𝑎𝑡𝑟𝑖𝑥

𝑠𝑖𝑧𝑒 𝑜𝑓 𝑝𝑙𝑎𝑖𝑛𝑡𝑒𝑥𝑡 𝑚𝑎𝑡𝑟𝑖𝑥 .

We now estimated the expansion rate of the encrypted data using experimental parameter setting and found it to be approximately 22.1 (Methods Section). Although we have initially mentioned expansion rate; the expansion rate does not immediately explain the difference between the network requirements of sPLINK and our secure protocol because the two methods use different approaches for performing GWAS. For instance, sPLINK uses central aggregator and compensator sites, which may increase its network requirements, there may also be different types of overhead in sPLINK that we are not aware of. Moreover, sPLINK uses a Newton- Raphson method that exchanges gradient and Hessian matrices to fit the model whereas our approach uses an IRLS-based approach where we divide null model fitting and p-value assignment steps. Our approach only shares encrypted matrices which are inflated in size by ciphertext expansion rates. Due to these factors, it is not exactly possible to tease out the differences and formulate the differences in network usage between the methods. We have now updated and clarified our justification (on page 9) about the differences in network transfers and explained above points on revised page 9:

“This difference can be partially explained by the fact that secure protocol transfers only encrypted data which are inflated in size by the ciphertext expansion rate (i.e., size of encrypted matrix divided by size of plaintext matrix), which is approximately 22.1 (Methods). There are, however, other factors that may impact differences. For example, sPLINK and our protocol use fundamentally different architectures (sPLINK uses central aggregator/compensator while our protocol relies on encrypted data exchanges from shared disk space) and methodology such as differences in model optimization (Newton-Raphson in sPLINK and IRLS in secure protocol) and estimation of p-values. The difference in network traffic is therefore a result of these factors.”

Also, Methods Section is updated to include estimate of expansion rate (Page 15):

“Ciphertext Expansion Rates Ciphertext expansion refers to inflation of size of ciphertext data compared to the underlying plaintext data. To estimate the ciphertext expansion rate for the GWAS protocol, we generated a random 64x128 matrix (full utilization of a ciphertext that can hold 8,192 entries) and encrypted it using COLLAGENE, and calculated the expansion rate as 𝑒𝑥𝑝𝑎𝑛𝑠𝑖𝑜𝑛 𝑟𝑎𝑡𝑒 = 𝑠𝑖𝑧𝑒 𝑜𝑓 𝑒𝑛𝑐𝑟𝑦𝑝𝑡𝑒𝑑 𝑚𝑎𝑡𝑟𝑖𝑥 𝑠𝑖𝑧𝑒 𝑜𝑓 𝑝𝑙𝑎𝑖𝑛𝑡𝑒𝑥𝑡 𝑚𝑎𝑡𝑟𝑖𝑥 . We repeated this estimation 100 times and found that the mean expansion rate is 22.1 with standard deviation of 0.08.”

R1.13. Our initial writing was not exact. When we used no networking setting (i.e., local shared directory), the whole protocol finished in 3,092 seconds. When we used AWS-based networked setting (i.e., AWS bucket is used as the shared directory), the protocol finished in 5,346 seconds. Thus, our estimate for the time requirement for networking is 5,346-3,092=2,254 seconds, i.e., 2,254/5,346=42% of the time is used for networking operations. This factor is now corrected to its exact value. We have updated and clarified the difference more accurately on revised page 9:

“We next assessed how much of the computation time is taken by network traffic. We ran our secure GWAS locally, i.e., all sites are simulated to share the same file system without any network exchanges. The overall protocol finished in 3,092 seconds (0.86 hours) wherein the model fitting required 877.8 seconds and the p-value assignment was completed in 2,214 seconds. When compared with networked protocol that finished in 5,346 seconds, this indicates that network transfers required 2,254 seconds (5,346 minus 3,092), approximately 42% of the computation time (2,254 seconds out network of 5,346 seconds of total time) for federated GWAS (A similar result was reported by sPLINK.)”

R1.14. Removed extra word “are” on revised page 14 in Discussion Section.

R1.15. Added Homer et al. reference on page 14 in Discussion Section.

R1.16. In original Figure 4, panels (e-h) showed the time and memory requirements for the base build of SEAL that is compiled into COLLAGENE. Original panels (i-l) show the requirements using the optimized build of SEAL (e.g., AVX optimizations). Please note that Figure 4(i-l) are now updated to make the differences more interpretable in new Figure 4 panels (g) and (h). Please see responses to R.17 and R.19 below. R1.17. We have now updated the original panels (i-l) of Figure 4 and replaced them with new panels (g) and (h). The new panels directly compare the time and memory usage of base and optimized builds of SEAL that is linked to COLLAGENE. We updated the legends accordingly. We hope that the new panels illustrate the comparisons more clearly.

R1.18. We removed the redundant legends from 4b and 4d, which are now moved to Figure 5a and 5c, respectively, in the revised manuscript.

R1.19. We updated Figure 4. We decided to keep the panels 4e-h in the revised panels 4c-f because they show detailed information about time and memory usage at different steps of the GWAS protocol. Original panels 4i-l have been replaced with new panels 4g and 4h. These panels illustrate the differences in time and memory usage of base and optimized build of COLLAGENE by plotting them explicitly on the bar plots. Please note that the panels corresponding to LOAD dataset results (original Fig. 4c, 4d) are now moved to Figure 5 and expanded with new panels.

We updated the results about comparison of base and optimized build of COLLAGENE on page 10: “The federated calculation completed the GWAS pipeline in 4,547 seconds (1.26 hours) with an optimized build of COLLAGENE. Of this total time, 1,652 seconds (0.46 hours) were spent for null model training, and 2,895 seconds were spent (0.80 hours) for p-value assignment (Fig. 4g). In comparison with the baseline build, we observed the main difference is at Step-6 where null model parameters were updated (Fig. 3a). This is expected as this step contains the most demanding HE operations (row-row matrix multiplications) that are optimized by the HEXL library. Similarly, the largest improvement for p-value assignment runtime was at Step-10 (Fig. 3b) where the sites pool the intermediate encrypted matrices (Fig. 4g). Peak memory usage of an optimized build of COLLAGENE was 1.1 gigabytes slightly higher (around 30 megabytes) than the baseline build (Fig. 4h). We also observed that the network I/O and storage were decreased in comparison to the baseline build. The optimized build of COLLAGENE used around 1.2 Gigabytes of network I/O and in total 3.6 gigabytes were used for storage at each client (Table 2). In summary, the optimizations in the underlying SEAL library make observable improvements in the performance.”

R1.20. We added Spearman R2 coefficients to new Figure 6a and 6b.

Reviewer #2

*Reviewer’s Comment: “The authors have introduced an integrated secured federated learning library that combines homomorphic encryption (HE) and differential privacy (DP) to enhance privacy preservation in federated training using GWAS data. Additionally, their proposal of an efficient GMMAT scoring method adds novelty to the methodology. In all, this study introduces a novel privacy- preserving federated learning scheme called COLLAGENE, which holds promise for various applications in different domains beyond GWAS data analysis. The framework's potential contribution extends to broader areas, making it an intriguing research endeavor..*

*Positives:*

*The authors have conducted a thorough investigation of state-of-the-art secured federated learning studies, highlighting their advantages and limitations. The utilization of null-model fitting and GMMAT scoring has demonstrated performance improvements compared to existing logistic regression models in the context of federated learning. Furthermore, the paper provides detailed optimization strategies for both software and hardware aspects, making the COLLAGENE framework efficient. The methodology is well-presented, and the framework is open source, which enhances the reproducibility of this work.”*

**Authors’ Response:** We thank the reviewer for constructive comments.

In this revision, we made the COLLAGENE repository publicly available. This repository contains the source  code, examples, use cases, and documentation (including command line interface specification and the  installation instructions). We made our best effort to make the codebase accessible and easy to build and run.

As requested by the reviewer, we have expanded on several results to clarify the conclusions. We also clarified  the aggregation process used in collective decryption and in the protocol design.

Our detailed responses are included below.

Reviewer’s Comment: “R2.1. It is commendable that the paper includes a detailed

analysis and comparison of the time and resource costs associated with the proposed

method. However, the manuscript can be improved by adding clarifications to their

methodology and results. For example, the manuscript would benefit from providing

explanations or formulas regarding the aggregation approach, and specifications

of the strategy employed for the aggregation process.”

Authors’ Response: We have gone over the methods sections and clarified following in more detail. Aggregation is used in numerous places in COLLAGENE. The most prevalent usage of aggregation is in the collective decryption protocol where sites aggregate partially decrypted matrices to calculate the fully decrypted matrix. We first describe the details of the collective decryption. We next describe the details of other places that aggregation such as intermediate statistics and column/row expansion of matrices. These are also included in the revised manuscripts (See below). Collective Decryption: Given an encrypted matrix, collective decryption generates the decrypted plaintext matrix, i.e., a plaintext matrix with double precision entries that can be readily used. When a single public/private key is used (e.g., SEAL), this is accomplished by SEAL’s native decryption interface using SEAL::Decryptor class. However, the private key (or secret key) does not exist in the multi-party key scenario that COLLAGENE addresses. Therefore, the collective decryption must use a new decryption algorithm that is build on top of the existing functionalities of SEAL’s Decryptor class. We describe the details of encryption and decryption operations below.

In the CKKS scheme, each ciphertext is represented as two polynomials, denoted by (𝑐0, 𝑐1). SEAL stores these in two arrays in the object of type SEAL::Ciphertext. Before encrypting a matrix, the entries are first encoded using an instance of the SEAL::CKKSEncoder class object, which generates a plaintext polynomial, 𝑚, that has the same size (number of terms) as ciphertext polynomials. It is worth noting that each public key in SEAL are ciphertexts that are generated by symmetric encryption of a zero polynomial, and they consist of two polynomials of the same size as the other ciphertexts. To be precise, given the public key polynomials, (𝑝𝑘0, 𝑝𝑘1), and the encoded plaintext polynomial of the matrix, 𝑚, the CKKS encryption calculates the two polynomials that make up the ciphertext. Let (𝑐0, 𝑐1) = 𝑒𝑛𝑐(𝑚, (𝑝𝑘0, 𝑝𝑘1)). The ciphertext is calculated as:

𝑐0 = 𝑝𝑘0 + 𝑚 (𝑚𝑜𝑑 𝑞), 𝑐1 = 𝑝𝑘1 (𝑚𝑜𝑑 𝑞)

The multiplications (additions) in this equation denote modular polynomial multiplications (additions) on the polynomial ring. These functions make use of number theoretic transform (NTT) representations to optimize polynomial multiplications and are implemented in SEAL. For polynomial operations, COLLAGENE relies on the polynomial arithmetic in SEAL and builds on top of these functions. Also, encryption is performed using the native SEAL interface (i.e., SEAL::Encryptor class) without any modifications. Of note, SEAL’s internal implementation of encryption is slightly different to include other random polynomials (Page 5 on [14]). We skip these for sake of simplicity. For decryption of a ciphertext (under single key scenario), the secret key is used. As a reminder, the secret key is a single polynomial sampled from the ternary distribution, i.e., {−1,0,1} 𝑚𝑜𝑑 𝑞. This key is generated by SEAL at the same time as the public key. By definition, decryption under the secret key 𝑠𝑘 is performed as:

𝑑𝑒𝑐(𝑐𝑡 = (𝑐0, 𝑐1); 𝑠𝑘) = (𝑐0 + 𝑐1 ⋅ 𝑠𝑘) 𝑚𝑜𝑑 (𝑞),

where 𝑑𝑒𝑐((𝑐0, 𝑐1); 𝑠𝑘) denotes the decryption of the ciphertext 𝑐𝑡. Combining encryption and decryption equations, the output of 𝑑𝑒𝑐((𝑐0, 𝑐1)) is (𝑚 + 𝑒), which is rounded to extract the original matrix polynomial, where 𝑒 is the total accumulated error in the ciphertext. As long as the accumulated error is small, the decryption can be correctly performed. This function is implemented in SEAL.

Details of Multi-Key Scenario: In the shared multi-party key scenario that COLLAGENE addresses, each site receives a secret key share, i.e., a noisy version of the secret key. The summation of the secret key shares, 𝑑𝑠𝑘𝑠, from all sites satisfy following:

𝑚𝑠𝑘 = (∑ 𝑑𝑠𝑘𝑠

𝑠

) 𝑚𝑜𝑑 (𝑞)

where summation is taken over the secret key polynomials and 𝑚𝑠𝑘 denotes the “master” secret key. (Of note, no site has access to the master secret key.) The master secret key is paired with the public key that KeyMaker generates and makes available for each site. Thus, any data that is encrypted with the COLLAGENE’s public key can be decrypted only with the master secret key. Following up from the formula for CKKS decryption, a ciphertext polynomial can be decrypted into the plaintext polynomial 𝑚 using the master secret key:

𝑑𝑒𝑐((𝑐0, 𝑐1); 𝑚𝑠𝑘) = 𝑒𝑛𝑐(𝑚, (𝑝𝑘0, 𝑝𝑘1)) = (𝑐0 + 𝑐1 ⋅ 𝑚𝑠𝑘) 𝑚𝑜𝑑 (𝑞).

Although no site has access to 𝑚𝑠𝑘, we can make use of 𝑑𝑠𝑘𝑠’s, which are available at each site, to calculate the decryption function as:

(𝑐0 + 𝑐1 ⋅ 𝑚𝑠𝑘) 𝑚𝑜𝑑 𝑞 = (𝑐0 + 𝑐1 ⋅ ∑ 𝑑𝑠𝑘𝑠

𝑠

) 𝑚𝑜𝑑 𝑞 = (𝑐0 + (𝑐1 ⋅ 𝑑𝑠𝑘1) + (𝑐1 ⋅ 𝑑𝑠𝑘2) + ⋯ + (𝑐1 ⋅ 𝑑𝑠𝑘𝑆) ) 𝑚𝑜𝑑 𝑞

We refer to each of the terms in parentheses, i.e., (𝑐1 ⋅ 𝑑𝑠𝑘𝑠), on the rightmost side as the “partial decryption” of the ciphertext by each site. We denote the partial decryption of the ciphertext (𝑐0, 𝑐1) by site 𝑠 as

𝑝𝑎𝑟𝑑𝑒𝑐((𝑐0, 𝑐1); 𝑑𝑠𝑘𝑠) = (𝑐1 ⋅ 𝑑𝑠𝑘𝑠) 𝑚𝑜𝑑 𝑞.

Note that the partial decryption is a single polynomial. Since all sites have access to the same ciphertext (they would like to decrypt it), and they have access to their own secret key shares 𝑑𝑠𝑘𝑠, they can calculate the partial decryption with a modular polynomial multiplication and share it with other sites. COLLAGENE implements the above partial decryption function using SEAL’s polynomial arithmetic interface. After all sites share their partial decryptions, 𝑝𝑎𝑟𝑡𝑑𝑒𝑐((𝑐0, 𝑐1); 𝑑𝑠𝑘𝑠), the final decryption is calculated by aggregating the partial decryptions by polynomial addition:

𝑑𝑒𝑐((𝑐0, 𝑐1); 𝑚𝑠𝑘) = 𝑐0 + ∑ (𝑝𝑎𝑟𝑑𝑒𝑐((𝑐0, 𝑐1); 𝑑𝑠𝑘𝑠)) 𝑚𝑜𝑑 (𝑞)

𝑠⏟

𝐴𝑔𝑔𝑟𝑒𝑔𝑎𝑡𝑖𝑜𝑛 𝑜𝑓 𝑝𝑎𝑟𝑡𝑖𝑎𝑙 𝑑𝑒𝑐𝑟𝑦𝑝𝑡𝑖𝑜𝑛𝑠 𝑏𝑦 𝑝𝑜𝑙𝑦𝑛𝑜𝑚𝑖𝑎𝑙 𝑎𝑑𝑑𝑖𝑡𝑖𝑜𝑛 𝑓𝑢𝑛𝑐𝑡𝑖𝑜𝑛 𝑖𝑛 𝑆𝐸𝐴𝐿.

The last remaining term 𝑐0 needs to be added only once to the final decryption (using polynomial addition) by one of the sites since it is known among all sites. In COLLAGENE, we implemented this in the partial decryption function where only the first site adds it once to its partially decrypted data. After aggregation, the final decrypted data is exactly equal to what sites would have decrypted if they had access to the master secret key.

Another important aspect is the protection of the partially decrypted data. In the above formulation, the partial decryptions are simple polynomial multiplications, (𝑐1 ⋅ 𝑑𝑠𝑘𝑠) 𝑚𝑜𝑑 𝑞. Any entity who has access to partial decryptions and the ciphertext (𝑐0, 𝑐1) can try to divide the partial decryption by 𝑐1 and recover the secret key share of site 𝑠. To provide protection against these attacks, COLLAGENE uses smudging noise, which is generated from the discrete Gaussian distribution implemented in SEAL library. Given a smudging noise variance in 𝑛𝑠𝑚 bits, i.e.,

𝜎𝑠𝑚

2 = 2𝑛𝑠𝑚 , a polynomial, denoted by 𝑒𝑠𝑚, is sampled from the clipped normal

distribution with variance 𝜎𝑠𝑚

2 and added to the partial decryption:

𝑝𝑎𝑟𝑑𝑒𝑐((𝑐0, 𝑐1); 𝑑𝑠𝑘𝑠) = ((𝑐1 ⋅ 𝑑𝑠𝑘𝑠) + 𝑒𝑠𝑚) 𝑚𝑜𝑑 (𝑞)

For an adversarial entity with access to 𝑐1 and the partial decryption, the goal will be to recover 𝑑𝑠𝑘𝑠 when 𝑒𝑠𝑚 is not known by the adversary. In the above equation, the exponentially large smudging noise statistically obliterates the information leakage from the partial decryption result, i.e., (𝑐1 ⋅ 𝑑𝑠𝑘𝑠). It should be noted that the partial decryptions should be protected from entities without authorization to access them. To accomplish this, COLLAGENE provides a symmetric key that can be used to encrypt the partially decrypted plaintext matrices. This key is shared among all sites.

Aggregation of Intermediate Statistics Matrices, Matrix Masks, and Row/Column Expansions: Aggregation of matrices with intermediate statistics and masks is performed using the ciphertext addition function over all the ciphertexts that make up the encrypted matrix. Similarly, row and column expansions are aggregated using ciphertext addition functions by looping over corresponding expansion terms.

Reviewer’s Comment: “R2.2. In the section of "Comparison on Real data", more details should be added to summarize the observations based on figures (e.g. Fig. 4.c and 4.d).”

Authors’ Response: In this revision, we expanded the analysis on LOAD dataset. Rather than focusing on the 60 thousand subsampled variants, we analyzed 557,056 variants (68 blocks of 8,192 variants such that each block exactly fits into one ciphertext) among 3 sites using the LOAD dataset. The genotype, phenotype, and covariates were randomly partitioned among 3 sites as in our original manuscript (848 subjects per site). To decrease network transfer cost and time, we used local file system as the shared encrypted data storage rather than using AWS bucket as we did in previous experiment.

We executed the secure and plaintext federated protocols on the distributed dataset and plink2 on the pooled dataset. For this revision, we also ran GMMAT on the pooled dataset to have a separate baseline for the significance levels.

P-value Comparisons: We first compared the p-values assigned to each variant by the protocols (Fig. 5a, b, c). The p-values assigned by plaintext-federated protocol and GMMAT are concordant to secure protocol (Spearman R2>0.99 for both comparisons), which indicates that secure protocol replicates the plaintext federated result and GMMAT’s pooled results with high concordance. Similar comparison between Plink2 and secure protocol showed a high concordance between these methods (Spearman R2>0.99). We focused on the variants that exhibit large differences between plink2 and secure federated protocol. Of note, we do not expect a perfect concordance because the score test has different statistical properties than plink2’s approach (e.g., score test asymptotically converges to the chi-squared distribution under null hypothesis). We specifically focused on the variant allele frequency and missingness as the possible source for differences. Concordant with GMMAT protocol, the secure protocol assigns mean imputed genotypes to each variant while plink2 excludes, for each SNP, the subjects with missing genotype. We observed that the SNPs with the high p-value difference exhibit high missingness (Fig 5d, e). This result shows that missingness may create differences between the results of secure protocol and plink2. We did not observe correlation between p-values differences of secure protocol and plink2 versus variant allele frequencies.

Manhattan Plots: We next evaluated the top SNPs identified as significantly associated with phenotype (i.e., AD diagnosis). Manhattan plots (Fig. 5f, g) shows that the most highly associated SNPs are located on 19q13.3, which is a known locus that is associated with AD status. We extracted the SNPs that pass the GWAS threshold (5x10-8) and found 9 SNPs that are identified by secure protocol, GMMAT, and plink2 (plaintext federated protocol gives the same SNPs as secure protocol) shown in Table 3. Overall, GMMAT and secure protocol assigned the same SNPs to be statistically significant. Among these variants, 8 of them were marked as significant by plink2. The remaining variant (rs4796606 on chr17:36917613) was assigned borderline p-value by plink2 at 5.38x10-8. GMMAT and secure federated protocol assigns similar p-value to this SNP.

REFERENCES

1. Paverd A, Martin A, Brown I. Modelling and automatically analysing privacy properties for honest-but- curious adversaries. [cited 31 May 2023]. Available:

https://www.cs.ox.ac.uk/people/andrew.paverd/casper/casper-privacy-report.pdf

2. Emam KE, Dankar FK, Vaillancourt R, Roffey T, Lysyk M. Evaluating the risk of re-identification of patients from hospital prescription records. Can J Hosp Pharm. 2009;62: 307–319.

3. Langer SG, Shih G, Nagy P, Landman BA. Collaborative and reproducible research: Goals, challenges, and strategies. J Digit Imaging. 2018;31: 275–282.

4. Sweeney L. Simple demographics often identify people uniquely. Carnegie Mellon University, Data Privacy Working Paper 3 Pittsburgh 2000. 2000.

5. Sweeney L, Abu A, Winn J. Identifying Participants in the Personal Genome Project by Name. SSRN Electronic Journal. 2013; 1–4.

6. Gymrek M, McGuire AL, Golan D, Halperin E, Erlich Y. Identifying personal genomes by surname inference. Science. 2013;339: 321–324.

7. Homer N, Szelinger S, Redman M, Duggan D, Tembe W, Muehling J, et al. Resolving individuals contributing trace amounts of DNA to highly complex mixtures using high-density SNP genotyping microarrays. PLoS Genet. 2008;4. doi:10.1371/journal.pgen.1000167

8. Sankararaman S, Obozinski G, Jordan MI, Halperin E. Genomic privacy and limits of individual detection in a pool. Nat Genet. 2009;41: 965–967.

9. Shringarpure SS, Bustamante CD. Privacy Risks from Genomic Data-Sharing Beacons. Am J Hum Genet. 2015;97: 631–646.

10. Sang Y, Shen H, Tian H. Effective reconstruction of data perturbed by random projections. IEEE Trans Comput. 2012;61: 101–117.

11. Liu K, Kargupta H, Ryan J. Random projection-based multiplicative data perturbation for privacy preserving distributed data mining. IEEE Trans Knowl Data Eng. 2006;18: 92–106.

12. Cristiani V, Lecomte M, Hiscock T, Maurine P. Fit the joint moments: How to attack any masking scheme. IEEE Access. 2022;10: 127412–127427.

13. Aumann Y, Lindell Y. Security against covert adversaries: Efficient protocols for realistic adversaries. Theory of Cryptography. Berlin, Heidelberg: Springer Berlin Heidelberg; 2007. pp. 137–156.

14. Laine K. Simple encrypted arithmetic library 2..1. [cited 1 Jul 2023]. Available:

https://www.microsoft.com/en-us/research/uploads/prod/2017/11/sealmanual-2-3-1.pdf

**2^nd^ round**

**Reviewer 1**

Thank you very much for the revision and the comprehensive explanation of all points in the letter. All of our points have been adressed and resolved.

**Reviewer 2**

The authors have made efforts to address the comments raised in my previous review.
